# Synergistic Modulation of Ru–O Bond Covalency via Ba/Fe Co‐Doping and Oxygen‐Vacancy Engineering for Efficient Wide‐pH Oxygen Evolution

**DOI:** 10.1002/advs.202520473

**Published:** 2026-02-16

**Authors:** Zongpeng Wang, Ting Jiang, Reem F. Alshehri, Longfei Ding, Salah M. El‐Bahy, Yuanmo Lin, Puyou Ying, Hongxiang Chen, Hanhui Lei, Terence X. Liu, Zhiping Lin

**Affiliations:** ^1^ College of Intelligent Manufacturing Putian University Putian China; ^2^ School of Material Science and Engineering Taizhou University Taizhou China; ^3^ Chemistry Department, College of Science Taibah University Madinah, 42353 Kingdom of Saudi Arabia; ^4^ Department of Chemistry, Turabah University College Taif University Taif Saudi Arabia; ^5^ School of Material Science and Engineering Fujian University of Technology Fuzhou China; ^6^ School of Engineering, Physics and Mathematics Northumbria University Newcastle upon Tyne United Kingdom

**Keywords:** co‐doping, covalency modulation, O vacancy, oxygen evolution reaction

## Abstract

Developing efficient and stable oxygen evolution reaction (OER) catalysts across a wide‐pH range is critical for practical water electrolysis. Here, it is demonstrated that co‐doping Ba and Fe into RuO_2_, together with oxygen vacancy engineering, synergistically tailors the Ru–O bond covalency, yielding highly active and durable OER catalysis across acidic, alkaline, and neutral media. Structural and spectroscopic analyses (XRD, XPS, and XAS) reveal that Ba doping expands the Ru–O bond length and weakens electron cloud overlap, whereas Fe doping contracts the bond and strengthens covalency. Their cooperative effect in co‐doped RuO_2_ produces a moderate Ru–O bond covalency, balancing adsorption energies of OER intermediates. Density functional theory further confirms that the neighboring Ru sites near dopants and oxygen vacancies provide optimal adsorption, lowering the rate‐limiting barrier. This covalency tuning strategy endows co‐doped RuO_2_ with both high intrinsic activity and structural stability, as evidenced by low overpotentials of 174, 236, and 284 mV at 10 mA cm^2^ in acidic, alkaline, and neutral electrolytes, and over 360 h of continuous operation with negligible degradation. This work highlights Ru–O bond covalency modulation as an effective design principle for wide‐pH, high‐performance Ru‐based OER catalysts.

## Graphical abstract

Ba/Fe co‐doping and oxygen vacancy engineering synergistically modulate the Ru–O bond covalency in RuO_2_, leading to markedly enhanced OER activity across wide‐pH electrolytes.

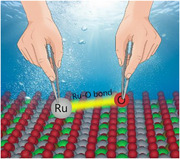

## 1 Introduction

The oxygen evolution reaction (OER) is a pivotal half‐reaction in many electrochemical energy conversion and storage technologies, including water electrolysis [1], rechargeable metal–air batteries [2], and CO_2_ reduction [3]. Despite its central importance, OER remains a major bottleneck owing to its sluggish four‐electron and four‐proton transfer pathway, which requires high overpotentials to overcome kinetic barriers [4]. This inefficiency severely limits the energy efficiency of electrochemical devices, underscoring the urgent need for advanced OER catalysts. Precious metal oxides, particularly RuO_2_ and IrO_2_, are recognized as benchmark OER catalysts due to their intrinsic activity and reasonable stability [5, 6]. However, several fundamental and practical challenges remain. First, the scarcity and high cost of Ru and Ir impede their scalability. Second, while RuO_2_ exhibits relatively higher activity than IrO_2_, it suffers from stability issues under harsh acidic and alkaline conditions, often undergoing Ru dissolution or structural degradation during long‐term operation [7, 8]. Third, even with these noble oxides, the catalytic activity is still insufficient to meet the stringent requirements of large‐scale hydrogen production and other industrial applications. Thus, rational design strategies are required to improve both the intrinsic activity and durability of RuO_2_‐based catalysts while simultaneously reducing their effective noble metal content.

To address these challenges, extensive efforts have focused on tailoring the physicochemical properties of RuO_2_ through heteroatom doping [7, 8, 9, 10, 11, 12, 13, 14], vacancy engineering [15, 16, 17, 18, 19, 20], nanostructure control [21, 22], and heterointerface construction [23, 24, 25, 26, 27]. Heteroatom doping introduces foreign cations into the RuO_2_ lattice, modulating its electronic structure and creating new active sites. For instance, transition‐metal dopants (e.g., Fe, Co, Mn, and V) have been shown to alter the electronic density of states near the Fermi level, optimizing the adsorption strength of key OER intermediates [28, 29]. Similarly, alkaline‐earth or rare‐earth dopants (e.g., Na, Sr, and La) have been explored to expand the lattice and modify oxygen coordination [30, 31, 32, 33]. Parallel to doping, defect engineering—especially the introduction of oxygen vacancies—has been employed to enhance conductivity, alter local coordination environments, and generate unsaturated active sites with improved reactivity. Nanostructure and morphology control, including creating nanoparticles, nanosheets, and hierarchical architectures [21, 22], further improve surface‐to‐volume ratios and exposure of catalytically active facets. While these approaches can modify the local electronic environment and create more active sites, the underlying design principle often remains empirical. In particular, the covalency of the Ru–O bond, which governs the binding strength of oxygen intermediates and directly influences OER kinetics, has rarely been tuned in a systematic and cooperative manner [34, 35, 36]. Excessively strong Ru–O bond covalency suppresses the interaction between Ru active sites and external oxygen species, thereby hindering their effective adsorption [33, 37]. In contrast, overly weak covalency leads to overly strong adsorption of oxygen intermediates and sluggish catalytic turnover. Achieving an optimal balance of intermediate Ru–O covalency, which requires precise modulation, represents a promising but underdeveloped strategy for designing highly active and stable OER catalysts.

Here, we demonstrate such a strategy through the co‐doping of Ba and Fe into RuO_2_, accompanied by oxygen vacancy engineering. Ba doping tends to elongate Ru–O bonds and weaken covalency, while Fe doping contracts bonds and strengthens covalency. Their cooperative interplay, coupled with oxygen vacancies, yields a moderate Ru–O bond covalency ideally suited for OER catalysis. Comprehensive characterization reveals the electronic and structural changes induced by Ba/Fe co‐doping and oxygen vacancies. Density functional theory calculations further uncover the mechanistic origins, showing that Ru sites adjacent to dopants and vacancies exhibit optimized adsorption free energies for OER intermediates. Electrochemical measurements confirm that co‐doped RuO_2_ achieves superior catalytic activity across acidic, alkaline, and neutral electrolytes, with overpotentials of 174, 236, and 284 mV at 10 mA cm^2^, respectively—significantly lower than pristine RuO_2_. In addition, the co‐doped RuO_2_ demonstrates excellent long‐term durability, maintaining stable activity for over 360 h with minimal performance loss. This study not only demonstrates a practical route toward wide‐pH OER catalysts but also highlights bond covalency engineering as a general design principle for tailoring the electronic structure of transition‐metal oxides.

## 2 Results and Discussion

**FIGURE 1:**
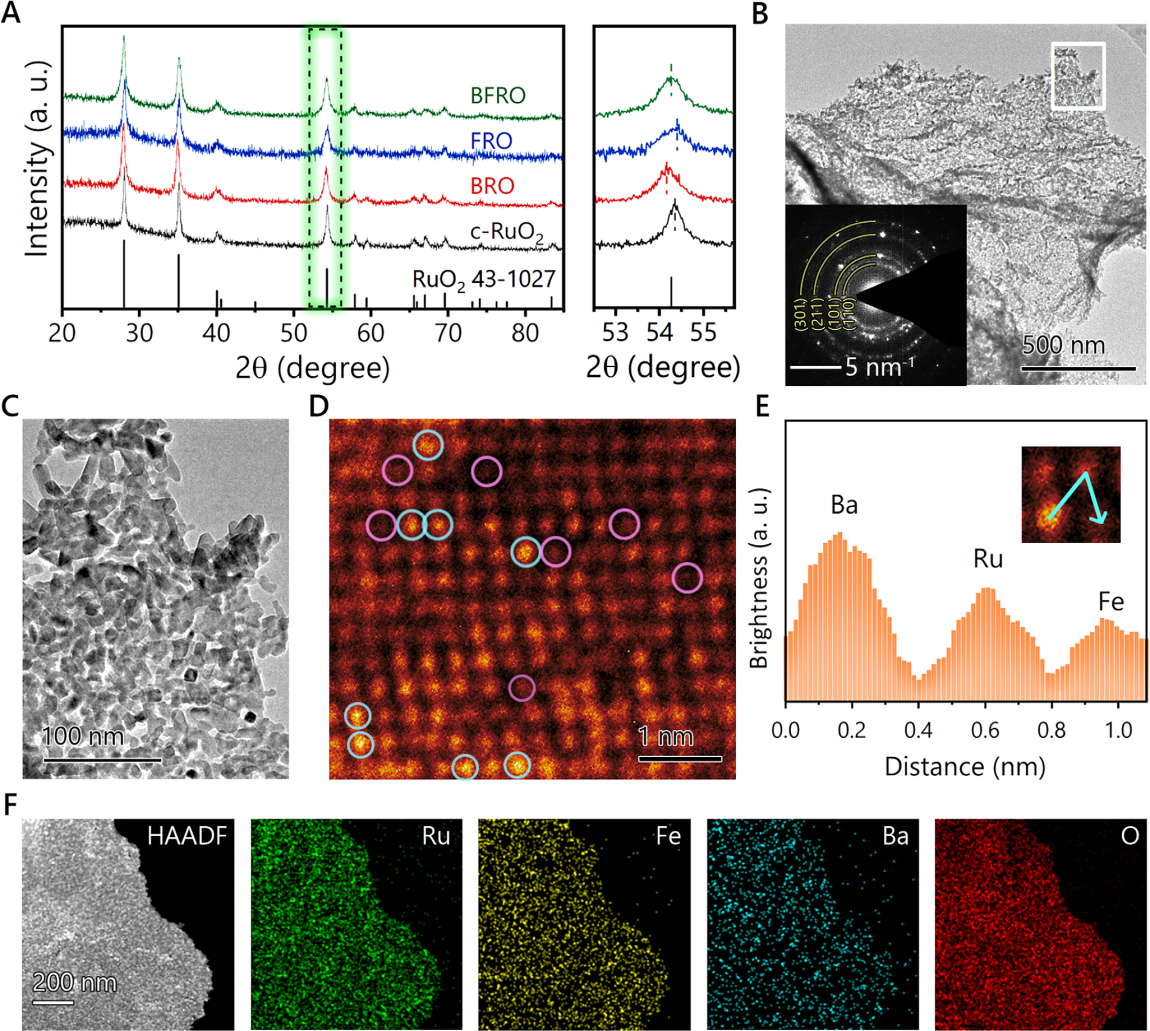
(A) Powder X‐ray diffraction patterns of RuO_2_, BRO, FRO, and BFRO. The enlarged view of the region highlighted by the dashed box is shown on the right. (B) TEM photo and SAED pattern (inset) of BFRO. (C) Magnified TEM photo of the region outline by a white rectangle in B. (D) Atom‐resolved HAADF‐STEM photo of BFRO. Representative Ba and Fe atoms are highlighted with blue and pink circles. (E) Line profile of brightness intensity along the inset arrow. (F) Element mapping of BFRO.

The RuO_2_‐based electrocatalysts were prepared by a one‐step hydrothermal cation exchange method (see details in supplementary information). Samples with nominal stoichiometric formula Ba_0.2_Ru_0.8_O_2‐_ *_x_*, Fe_0.2_Ru_0.8_O_2‐_ *_x_* and Ba_0.1_Fe_0.1_Ru_0.8_O_2‐_ *_x_* are denoted as BRO, FRO and BFRO, for convenience. The dopant contents are estimated by inductively coupled plasma (ICP) measurements, with results summarized in Table S2, which are in good agreement with their nominal contents. Figure 1A presents the PXRD (powder X‐ray diffraction) patterns of pristine RuO_2_ and its doped counterparts. All diffraction peaks are well indexed to the rutile RuO_2_ phase (JCPDS No. 43–1027; see Table S1 for details), with no additional peaks observed, confirming the phase purity and reliability of the synthesis method. The successful incorporation of dopants is evidenced by the slight shifts in diffraction peaks. For instance, the peak associated with the (211) crystal plane exhibits a noticeable shift, as shown in the enlarged view. This shift can be attributed to the difference in atomic radii between the dopants and Ru. Specifically, Ba atoms, being larger than Ru, expand the crystal lattice and causes a shift toward lower diffraction angles, whereas the smaller Fe atoms induce lattice contraction, leading to a shift toward higher angles. Such lattice distortions modulate the Ru–O bond length, thereby enabling the fine‐tuning of Ru–O bond covalency, which will be further discussed below.

Figure 1B shows the TEM (transmission electron microscopy) image of BFRO, in which a film‐like morphology is evident. SEM images (Figure S1) confirm that all prepared samples exhibit similar film‐like morphology. To investigate the fine structural features, the region outlined by the white rectangle is enlarged in Figure 1C, revealing that the film is composed of numerous small nanoparticles. The corresponding SAED (selected area electron diffraction) pattern, displayed in the inset of Figure 1B, exhibits multiple diffraction rings, which can be indexed to the (110), (101), (211), and (301) crystal plane families of RuO_2_, respectively. These results confirm the polycrystalline nature of the sample, consistent with the observed nanoparticle morphology. The presence of heteroatom dopants in BFRO is further verified by atom‐resolved HAADF‐STEM imaging (Figure 1D). In HAADF‐STEM, heavier atoms scatter incident electrons more strongly, appearing brighter in the image. As shown in Figure 1D, three distinct brightness levels can be clearly distinguished, corresponding to Ba, Ru, and Fe atoms, respectively. Representative Ba and Fe atoms are highlighted with blue and pink circles. To explicitly confirm the incorporation of heteroatom dopants, the brightness intensity profile along the arrow‐marked line (inset) is shown in Figure 1E, where the contiguous distribution of Ba, Ru, and Fe atoms is evident. Moreover, additional regions exhibiting lattice fringes of BFRO are presented in Figures S2 and S3. The measured fringe spacing of 3.118 Å corresponds well with the interplanar distance of the (110) crystal plane of RuO_2_. The presence of Ba and Fe dopants is further evidenced by variations in the intensity of the lattice fringes. Additionally, the elemental mapping is conducted with results presented in Figure 1F, which demonstrates the uniform distribution of Ba, Fe, Ru, and O elements throughout the sample, further confirming the successful synthesis of Ba/Fe‐doped RuO_2_.

**FIGURE 2:**
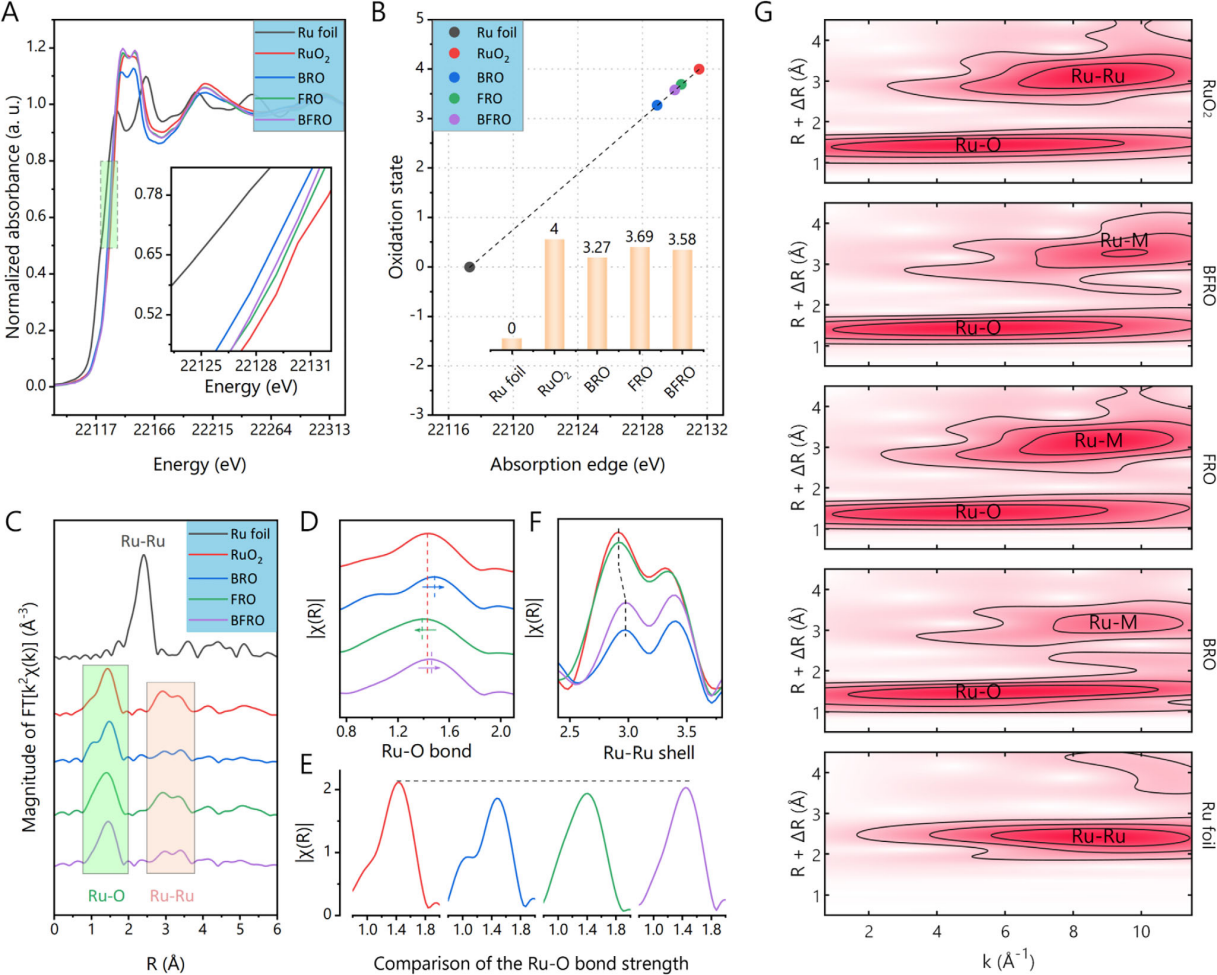
(A) Normalized Ru *K*‐edge XANES of RuO_2_, BRO, FRO and BFRO, respectively. Enlarged view of the absorption edge is shown in the inset. (B) Valence fitting of Ru element in corresponding samples according to the Ru *K*‐edge XANES data. (C) Ru *K*‐edge FT‐EXAFS spectra of RuO_2_, BRO, FRO and BFRO, respectively. (D, E) Comparison of the length and strength of Ru‐O bonds in RuO_2_, BRO, FRO and BFRO, respectively. (F) Comparison of the Ru‐Ru shell in RuO_2_, BRO, FRO and BFRO, respectively. (G) Wavelet transform of the Ru *K*‐edge EXAFS data of RuO_2_, BRO, FRO and BFRO, respectively.

The oxidation state and coordination environment of Ru in the samples were investigated by X‐ray absorption spectroscopy (XAS). Figure 2A presents the normalized Ru K‐edge XANES spectra, with an enlarged view of the absorption edge region shown in the inset. The absorption edges of BRO, FRO, and BFRO lie between those of Ru foil and RuO_2_, suggesting Ru valence states in these samples are in the range of 0 to +4. The absorption edge positions, determined from the first derivative of the normalized XANES spectra (Figure S4), follow the order of BRO < BFRO < FRO. Quantitative analysis by linear fitting with Ru foil and RuO_2_ standards (Figure 2B) yields average Ru valence states of 3.27, 3.69, and 3.58 for BRO, FRO, and BFRO, respectively. These results demonstrate that both Ba and Fe doping lower the oxidation state of Ru, with Ba exerting a stronger effect than Fe. Since electron‐rich Ru sites are known to enhance catalytic activity but compromise stability, Ba/Fe co‐doping is expected to provide a balance between activity and durability.

The k‐space EXAFS spectra are shown in Figures S5 and S6, and the corresponding Fourier transforms are presented in Figure 2C and Figures S7 and S8. As illustrated in Figure 2C, the peak at ∼1.4 Å is attributed to the Ru–O bond, whereas the peak near ∼3.0 Å corresponds to Ru–Ru scattering path. Since the electronic properties and catalytic activity of Ru sites in RuO_2_‐based electrocatalysts are strongly governed by the Ru–O bond, a detailed analysis on the Ru‐O bond was conducted. As shown in Figure 2D, compared to pristine RuO_2_, Ba doping leads to elongation of the Ru–O bond, while Fe doping results in bond shortening. This observation is consistent with the XRD results. As a comprehensive consequence, the average Ru–O bond length in BFRO is slightly longer than in RuO_2_. Given that Ru–O covalency is closely correlated with bond length, Ba/Fe doping enables its fine modulation. Furthermore, the bond strength follows the order BRO < BFRO < FRO < RuO_2_ (Figure 2E), suggesting that doping induces oxygen vacancies, with Ba exerting the more pronounced effect. The coexistence of oxygen vacancies and elemental doping alters the Ru–O orbital overlap, thereby tuning the covalency of the Ru–O bond from another aspect. It is also worth noting that Ba doping significantly reduces the Ru–Ru coordination number while simultaneously increasing the Ru–Ru coordination length (Figure 2F). This suggests that the incorporation of Ru not only weakens the electronic cloud overlap between the central Ru atom and its nearest‐neighbor O atoms, but also expands the Ru lattice framework. Both ways suppress the delocalized motion of electrons and thus reduce the covalency of the Ru–O bonds. The wavelet transform of the Ru K‐edge EXAFS data is conducted and shown in Figure 2G, where scattering atoms can be distinguished according to their mass in *k* axis. The significant right shift of Ru‐Metal center for BRO and BFRO clearly signifies the incorporation of Ba.

**FIGURE 3:**
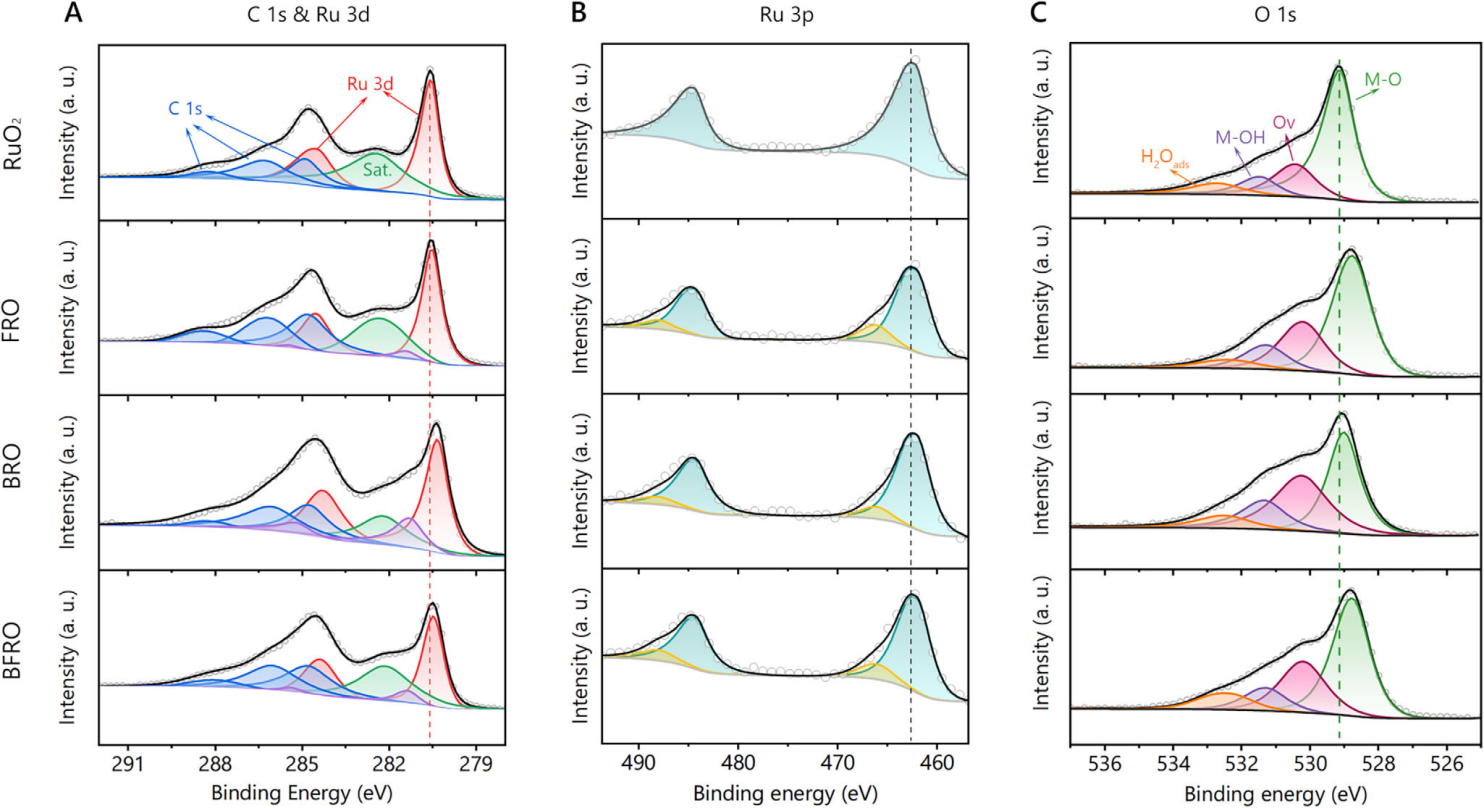
(A) High‐resolution C 1s and Ru 3d XPS spectra of RuO_2_, BRO, FRO and BFRO, respectively. (B) High‐resolution Ru 3p XPS spectra of RuO_2_, BRO, FRO and BFRO, respectively. (C) High‐resolution O 2p XPS spectra of RuO_2_, BRO, FRO and BFRO, respectively.

Lattice distortion and heteroatom doping induce charge redistribution along the Ru–O bond, which was examined by X‐ray photoelectron spectroscopy (XPS). The full survey spectra of all prepared samples (Figures S9–S12) show only Ru and O signals for pristine RuO_2_, additional Ba or Fe signals for BRO and FRO, and both Ba and Fe signals for BFRO, confirming the high purity of the samples. The high‐resolution Ru 3d spectra are presented in Figure 3A. Two sets of peaks were deconvoluted. The blue peaks correspond to the C 1s orbital, attributed respectively to C–C (284.8 eV), C–O (286.2 eV), and C = O (288.4 eV) bonds. The three residual peaks can be assigned to Ru 3d_5/2_ (280.6 eV), Ru 3d_3/2_ (284.6 eV), and a satellite feature. The Ru binding energies follow the order RuO_2_> FRO > BFRO > BRO, suggesting that both Ba and Fe act as electron reservoirs, donating electrons to Ru via the M–O–Ru bridge, as further supported by theoretical calculations. The high‐resolution Ru 3p spectra (Figure 3B) exhibit similar chemical shifts to Ru 3d. Furthermore, two peaks at 281.3 eV (3d orbital) and 466.3 eV (3p orbital) corresponding to Ru^3+^ are observed in the doped samples, suggesting the presence of O vacancies. Notably, Ba doping results in a more pronounced Ru^3+^ signal compared with Fe. The presence of oxygen vacancies is further corroborated by the O 1s spectra (Figure 3C), which can be deconvoluted into four contributions, namely, M–O bonds, oxygen vacancies (Ov), M–OH bonds, and adsorbed H_2_O. The Ov/M–O area ratios are 0.29, 0.89, 0.47, and 0.52 for RuO_2_, BRO, FRO, and BFRO, respectively, consistent with the Ru^3+^ peak intensities. Electron paramagnetic resonance (EPR) measurements were also conducted to verify the presence of oxygen vacancies, as shown in Figure S13. All samples display clear signals associated with oxygen vacancies, with intensities following the order RuO_2_< FRO < BFRO < BRO, which is consistent with the XPS results. Additionally, the M–O binding energies decrease in the doped samples relative to RuO_2_, indicating electron‐rich O atoms. Taken together, the XPS results suggest that electron transfer from Ba/Fe dopants to O weakens the Ru–O interaction, thereby reducing the covalency of the Ru–O bond.

**FIGURE 4:**
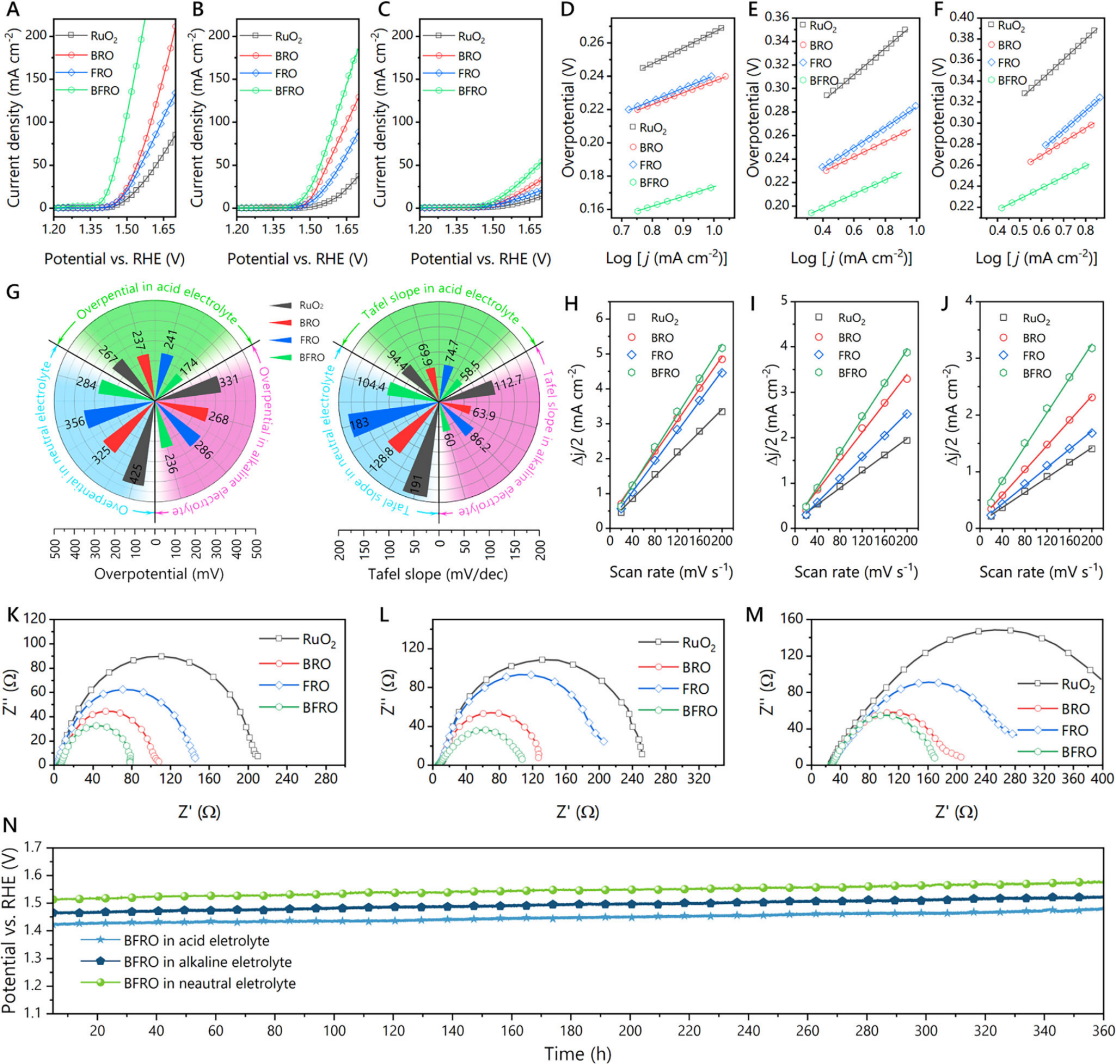
(A–C) LSV curves of RuO_2_, BRO, FRO, and BFRO measured in acidic (0.5 M H_2_SO_4_), alkaline (1 M KOH) and neutral (mixed solution of 1 M K_2_HPO_4_ and 1 M KH_2_PO_4_) electrolytes, respectively. (D–F) Tafel plots of RuO_2_, BRO, FRO and BFRO measured in acid measured in acidic, alkaline, and neutral electrolytes, respectively. (G) Summary diagram of the overpotential at 10 mA cm^2^ and the Tafel slopes of corresponding samples in acidic, alkaline, and neutral electrolytes. (H–J) C_dl_ plots of RuO_2_, BRO, FRO and BFRO derived from CV sweeps measured in acidic, alkaline, and neutral electrolytes, respectively. (K–M) Nyquist plots RuO_2_, BRO, FRO and BFRO measured in acid measured in acidic, alkaline, and neutral electrolytes, respectively. (N) Chronopotentiometric curves of BFRO measured in acidic, alkaline, and neutral electrolytes, respectively.

The electrocatalytic OER activity of the prepared samples was systematically evaluated in a wide pH range using 0.5 M H_2_SO_4_ (acidic), 1 M KOH (alkaline), and a neutral phosphate buffer (1 M K_2_HPO_4_/1 M KH_2_PO_4_) as electrolytes. The linear sweep voltammetry (LSV) curves (Figure 4A–C) and corresponding Tafel plots (Figure 4D–F) consistently demonstrate that the Ba/Fe co‐doped sample BFRO exhibits markedly superior performance compared to the other samples. The overpotentials at 10 mA cm^−2^ and the Tafel slopes are summarized in Figure 4G and Table S2. Specifically, BFRO delivers overpotentials of 174, 236, and 284 mV in acidic, alkaline, and neutral media, respectively, significantly lower than those of pristine RuO_2_ (267, 461, and 425 mV). Likewise, the Tafel slopes of BFRO (58.5, 60.0, and 104.4 mV dec^−1^) are much smaller than those of RuO_2_ (94.4, 112.7, and 191 mV dec^−1^), evidencing faster reaction kinetics enabled by Ru–O covalency modulation. The overpotential at 10 mA cm^−2^ are compared with other RuO_2_‐based catalysts in Table S5. To further probe the origin of the enhanced OER activity, the electrochemical surface area (ECSA) was estimated from the double‐layer capacitance (C_dl_) values (Figure 4H–J; Figures S14–S16). The specific ECSA values are summarized in Table S4. BFRO exhibits the largest ECSA across all electrolytes, suggesting a higher density of accessible active sites. Moreover, the ECSA normalized LSV curves are plotted in Figures S17–S19, which reveals BFRO exhibits the best intrinsic activity. Electrochemical impedance spectroscopy (EIS) measurements were conducted to evaluate the charge transfer properties (Figure 4K–M). Fitting of the Nyquist plots using an equivalent circuit model reveals that BFRO has the lowest charge‐transfer resistance, attributed to the presence of oxygen vacancies and improved conductivity. Stability, a key metric for practical application, was assessed by chronopotentiometry at a constant current density of 10 mA cm^−2^ (Figure 4N). BFRO demonstrates excellent durability, maintaining its activity with less than 3% performance loss over 360 h in all tested electrolytes. XRD, TEM and EDS measurements of the BFRO catalyst after the long‐term stability test were conducted, as shown in Figures S20–S23. The results demonstrate that BFRO preserves its morphology, crystal structure, and elemental composition after prolonged operation, indicating excellent structural stability. Ba/Fe doping is expected to influence Ru dissolution resistance through dopant‐dependent stabilization of the Ru–O framework, which is closely related to Ru over‐oxidation. Ba doping induces lattice expansion and Ru–O bond elongation, weakening Ru–O covalency and suppressing excessive charge delocalization, thereby mitigating the propensity of Ru to reach highly oxidized, dissolution‐prone states. In contrast, Fe doping slightly strengthens Ru–O interactions by enhancing Ru 3d–O 2p hybridization (see the calculation results below), which can reinforce the octahedral framework and hinder rapid lattice reconstruction at oxidizing potentials. Importantly, co‐doping balances these effects, yielding a moderate Ru–O covalency that simultaneously limits Ru over‐oxidation and preserves structural integrity. Consistent with this cooperative stabilization, BFRO exhibits negligible activity decay during prolonged operation, supporting enhanced resistance to dissolution under harsh OER conditions. These results collectively highlight the efficacy of the Ba/Fe co‐doping and Ru–O covalency tuning strategy in achieving high activity, fast kinetics, and long‐term stability across a wide pH range.

**FIGURE 5:**
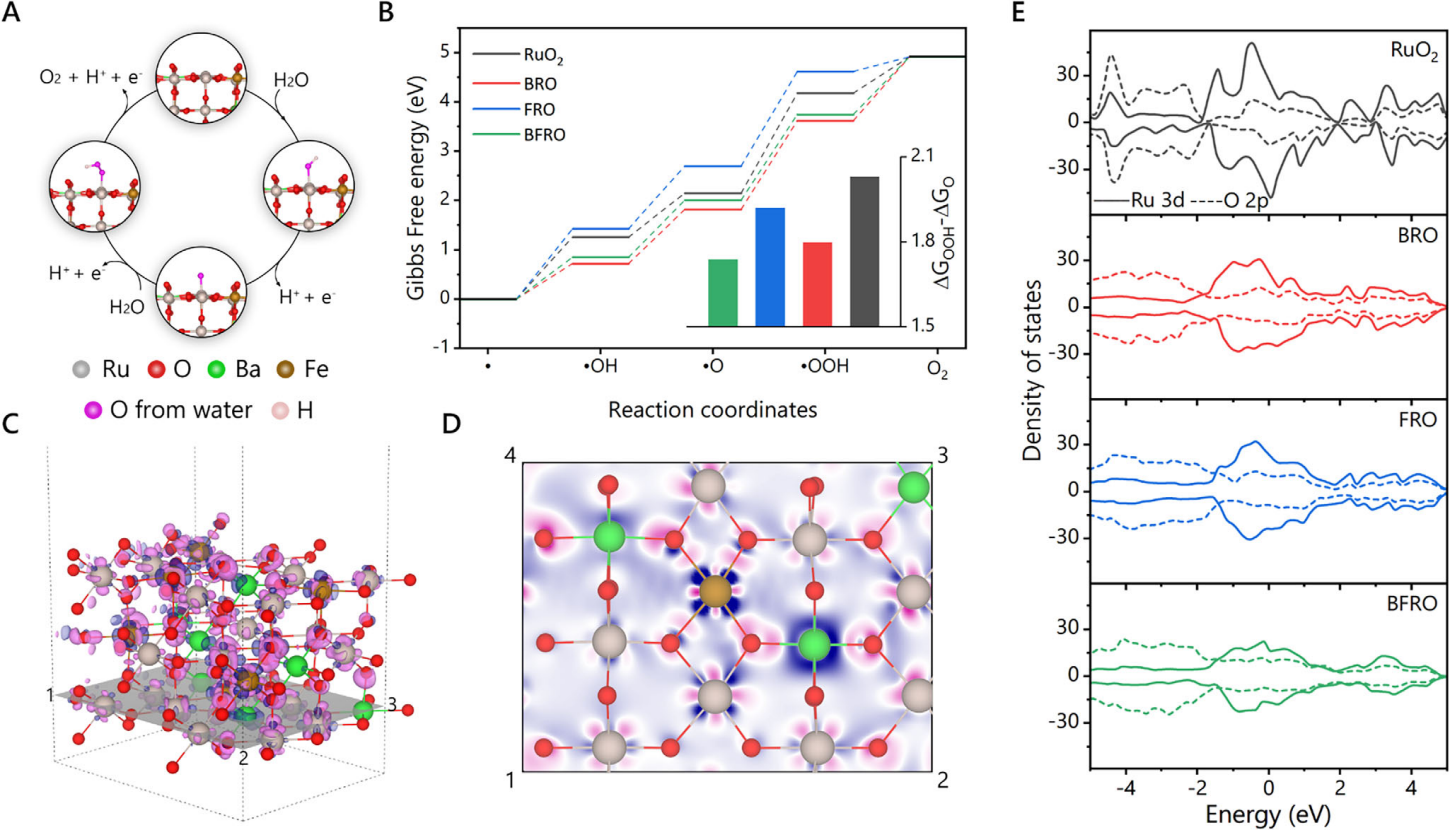
(A) Mechanism diagram of the OER process on BFRO in acidic electrolyte. (B) Gibbs free energy change during OER. (C) Differential charge density of BFRO. (D) Differential charge density on the corss‐secion “1‐2‐3‐4” in (C). (E) Density of states of the Ru 3d and O 2p orbitals of corresponding samples.

To elucidate the underlying mechanisms induced by Ba/Fe co‐doping and oxygen vacancies, density functional theory (DFT) calculations were performed. The computational models were constructed based on the (110) crystal plane of RuO_2_ (Figures S24–S27), in accordance with previous reports. Oxygen vacancies were introduced by removing four O atoms, while 20% of the Ru atoms were randomly substituted by Ba or Fe. The OER pathways (Figure 5A; Figures S30–S33) were examined to illustrate the cooperative effects of Ba/Fe co‐doping and oxygen vacancies. The results reveal that Ru sites adjacent to dopants exhibit the highest OER activity, underscoring the synergistic effect. The calculated Gibbs free energy profiles of reaction intermediates (Figure 5B; Figures S28 and S29) indicate that the transition from •O to •OOH adsorption constitutes the rate‐limiting step across all models. For RuO_2_ with oxygen vacancies, •O adsorption is excessively strong, whereas •OOH adsorption is insufficient. Ba doping enhances •OOH adsorption but further strengthens •O adsorption due to the linear scaling relationship. In contrast, Fe doping markedly weakens adsorption, thereby impeding intermediate capture at active sites. When combined, the compensating effects of Ba and Fe co‐doping yield moderate adsorption strength, effectively lowering the rate‐limiting barrier (inset of Figure 5B).

Differential charge density analysis further clarifies the origin of the enhanced catalytic performance (Figure 5D,E; Figures S34–S36). Both Ba and Fe dopants facilitate electron transfer to Ru through M–O–Ru bridges, consistent with XAS and XPS characterizations. Nevertheless, their charge redistribution behaviors differ substantially. Ba doping distorts the electron cloud around Ru, reducing electron density along the Ru–O bond axes while increasing density along the symmetry line between adjacent Ru–O bonds (Figure S35). This distortion diminishes Ru–O orbital overlap and strengthens the interaction between Ru and adsorbed O intermediates. Conversely, Fe doping increases electron density along the Ru–O direction (Figure S36), enhancing orbital overlap and thereby weakening Ru–intermediate interactions. These contrasting effects are corroborated by density of states analyses (Figure 5E), where Ru 3d–O 2p orbital overlap near the Fermi level is weakened in BRO but reinforced in FRO. In summary, the opposing influences of Ba and Fe doping can be rationalized by their modulation of Ru–O bond covalency. Ba doping elongates Ru–O bonds (as verified by XRD and EXAFS) and reduces orbital overlap, thereby weakening covalency, whereas Fe doping shortens bond lengths and increases overlap, strengthening covalency. Their cooperative modulation yields an intermediate covalency in BFRO, enabling optimal adsorption energetics and superior OER performance.

## 3 Conclusions

In summary, Ba and Fe dopants, together with oxygen vacancies, were successfully incorporated into RuO_2_ via a facile hydrothermal approach. The optimized BFRO catalyst exhibits low overpotentials of 174, 236, and 284 mV to achieve a current density of 10 mA cm^−2^ in acidic, alkaline, and neutral electrolytes, respectively. In addition, BFRO demonstrates remarkable durability, maintaining continuous operation for over 360 h with less than 3% performance degradation at 10 mA cm^−2^ across the wide‐pH range. Structural and electronic characterizations (XRD, XPS, and XAS) indicate that Ba/Fe co‐doping not only induces crystal distortion but also modulates the space‐charge distribution within the Ru–O framework, thereby effectively tuning Ru–O bond covalency. Complementary DFT calculations further reveal that co‐doping optimizes the adsorption energies of key OER intermediates, reduces the rate‐limiting energy barrier, and imparts enhanced catalytic activity to Ru sites adjacent to dopants and vacancies. This work highlights covalency tuning as a powerful strategy for designing robust Ru‐based catalysts with wide‐pH OER applicability.

## Conflicts of Interest

The authors declare no conflicts of interest.

## Supporting information




**Supporting File**: advs74429‐sup‐0001‐SuppMat.pdf

## Data Availability

The data that support the findings of this study are available from the corresponding author upon reasonable request.
