# Supplementary material for: Synergistic Modulation of Ru–O Bond Covalency via Ba/Fe Co‐Doping and Oxygen‐Vacancy Engineering for Efficient Wide‐pH Oxygen Evolution
Source: Adv Sci (Weinh). 2026 Feb 16;13(24):e20473. doi: 10.1002/advs.202520473 (PMC13116127; doi:10.1002/advs.202520473)
Supplement: Supplementary file 1 — Supporting File: advs74429‐sup‐0001‐SuppMat.pdf [file ADVS-13-e20473-s001.docx]

Supporting Information

1. Experiments
   1. Chemicals

Glucose (C_6_H_12_O_6_) and urea (CO(NH_2_)_2_) were purchased from Sinopharm Chemical Reagent Co., Ltd. Barium nitrate (Ba(NO_3_)_2_) and iron(III) chloride hexahydrate (FeCl_3_·6H_2_O) were obtained from Aladdin Reagent Co., Ltd. Ruthenium(III) chloride hydrate (RuCl_3_∙nH_2_O) was supplied by Macklin Biochemical Co., Ltd. All chemical reagents were used as received without further purification.

- 1. Preparation of RuO_2_.

To prepare the RuO_2_ sample, 33.4 mmol of glucose and 20 mmol of urea (CO(NH_2_)_2_) were dissolved in 20 mL of deionized water to form a homogeneous solution. Subsequently, 0.2 mmol of RuCl_3_·nH_2_O was added to the solution. After thorough mixing of all components, the mixture was transferred into a polytetrafluoroethylene-lined autoclave and heated to 160 °C in an oven for 10 hours. The product obtained after the hydrothermal reaction was calcined in air at 400 °C for 12 hours to yield RuO_2_

- 1. Preparation of BRO.

To synthesize Ba-doped RuO_2_ (B-RuO_2_), 33.4 mmol of glucose and 20 mmol of urea were dissolved in 20 mL of deionized water to obtain a homogeneous solution. Then, 0.16 mmol of RuCl_3_·nH_2_O and 0.04 mmol of Ba(NO_3_)_2_ were added to the solution. After thorough mixing, the resulting mixture was transferred to a polytetrafluoroethylene-lined autoclave and heated at 160 °C for 10 hours. The product obtained after the hydrothermal process was calcined in air at 400 °C for 12 hours, and the final product was referred to as BRO.

- 1. Preparation of FRO.

For the preparation of the Fe-doped RuO_2_ (F-RuO_2_) sample, 33.4 mmol of glucose and 20 mmol of urea were dissolved in 20 mL of deionized water to form a homogeneous solution. Then, 0.16 mmol of RuCl_3_·nH_2_O and 0.04 mmol of FeCl_3_·6H_2_O were added to the solution. After ensuring uniform mixing, the solution was transferred into a polytetrafluoroethylene-lined autoclave and heated at 160 °C for 10 hours. The resulting product from the hydrothermal reaction was calcined in air at 400 °C for 12 hours to yield the final product, designated as FRO.

- 1. Preparation of BFRO.

To prepare the RuO_2_-BaFe sample, 33.4 mmol of glucose (C_6_H_12_O_6_) and 20 mmol of urea (CO(NH_2_)_2_) were dissolved in 20 mL of deionized water to form a homogeneous solution. Subsequently, 0.16 mmol of RuCl_3_∙nH_2_O, 0.02 mmol of Ba(NO_3_)_2_, and 0.02 mmol of FeCl_3_·6H_2_O were added to the solution. The mixture was stirred thoroughly to ensure uniform dispersion of all the components. The resulting solution was then transferred into the liner of a polytetrafluoroethylene (PTFE)-lined autoclave. The autoclave was heated to 160 °C in an oven and maintained at this temperature for 10 hours. After the hydrothermal reaction, the obtained sample was calcined in air at 400 °C for 12 hours to yield the final product, designated as BFRO.

1. Methods
   1. Characterization

X-ray diffraction (XRD) patterns were recorded on a Bruker D8 Advance diffractometer equipped with Cu Kα radiation. Scanning electron microscopy (SEM) images were obtained using a Hitachi S-4800 instrument. Transmission electron microscopy (TEM), scanning transmission electron microscopy (STEM), selected area electron diffraction (SAED), and energy-dispersive X-ray spectroscopy (EDX) analyses were performed on a spherical-aberration-corrected JEOL JEM-ARF2100F microscope. X-ray photoelectron spectroscopy (XPS) measurements were carried out on a Thermo Fisher Scientific ESCALAB 250Xi spectrometer with an Al Kα radiation source (hν = 1486.6 eV).

- 1. Electrochemical measurements

Electrochemical measurements were conducted on a CHI 660E workstation using a three-electrode configuration at 30 °C. Linear sweep voltammetry (LSV) was performed at a scan rate of 5 mV/s, and all potentials were corrected for iR drop. Cyclic voltammetry (CV) was carried out within the potential window of 1.0–1.1 V versus the reversible hydrogen electrode (RHE), with the scan rate progressively increased from 20 to 200 mV/s. Electrochemical impedance spectroscopy (EIS) was measured at a static potential of 1.4 V over the frequency range from 100 kHz to 0.1 Hz.

The electrochemically active surface area (ECSA) was estimated based on the double-layer capacitance (C_dl_). Specifically, the ECSA was calculated using the equation ECSA = C_dl_/C_s_, where C_s_ represents the specific capacitance of a flat electrode surface. For acidic, neutral, and alkaline electrolytes, C_s_ values of 0.06 mF cm^-2^ (0.5 M H_2_SO_4_), 0.04 mF cm^-2^ (1 M PBS buffer), and 0.04 mF cm^-2^ (1 M KOH), respectively, were adopted.

- 1. DFT calculations

Density functional theory (DFT) calculations were performed using the Vienna Ab Initio Simulation Package (VASP) within the framework of the generalized gradient approximation (GGA) employing the Perdew–Burke–Ernzerhof (PBE) functional. The projector augmented-wave (PAW) method was used to describe the interaction between ionic cores and valence electrons, with a plane-wave basis set and a kinetic energy cutoff of 450 eV. Gaussian smearing with a width of 0.1 eV was applied to determine partial occupancies of the Kohn–Sham orbitals. The electronic self-consistency criterion was set to an energy convergence threshold of 10^-5^ eV, and geometry optimizations were deemed converged when the residual force on each atom was below 0.05 eV Å^-1^. Dispersion interactions were accounted for using Grimme’s DFT-D3 correction scheme. Brillouin zone integrations were performed with a Monkhorst–Pack k-point mesh of 3 × 3 × 1. A Hubbard U correction of 4.8 eV was applied to Ru atoms. The free energy was evaluated according to G=Eads+ZPE−TS, where G, Eads, ZPE and TS denote the free energy, total energy from DFT calculations, zero-point energy, and entropic contribution, respectively.

1. Supplementary Figures and Tables

Table S1. Lattice details of PDF card No. 43-1027, namely RuO_2_.

| 2θ (degree) | Intensity | Face spacing (Å) | Face index |
| --- | --- | --- | --- |
| 28.018 | 100 | 3.182 | (110) |
| 35.065 | 77 | 2.557 | (101) |
| 40.04 | 19 | 2.25 | (200) |
| 40.547 | 5 | 2.223 | (111) |
| 45.02 | 1 | 2.012 | (210) |
| 54.266 | 54 | 1.689 | (211) |
| 57.921 | 13 | 1.5908 | (220) |
| 59.45 | 7 | 1.5535 | (002) |
| 65.556 | 11 | 1.4228 | (310) |
| 65.91 | 1 | 1.416 | (221) |
| 66.978 | 12 | 1.396 | (112) |
| 69.54 | 15 | 1.3507 | (301) |
| 73.09 | 1 | 1.2936 | (311) |
| 74.103 | 6 | 1.2784 | (202) |
| 76.233 | 1 | 1.2479 | (320) |
| 77.57 | 1 | 1.2297 | (212) |
| 83.392 | 9 | 1.158 | (321) |
| 86.433 | 3 | 1.1249 | (400) |
| 87.738 | 7 | 1.1115 | (222) |
| 89.794 | 1 | 1.0913 | (410) |

Table S2. Ba and Fe content determined by ICP-OES.

| Sample | Fe/Ru mass ratio (%) | Ba/Ru mass ratio (%) |
| --- | --- | --- |
| BRO | / | 25.6 |
| FRO | 12.4 | / |
| BFRO | 5.9 | 11.9 |


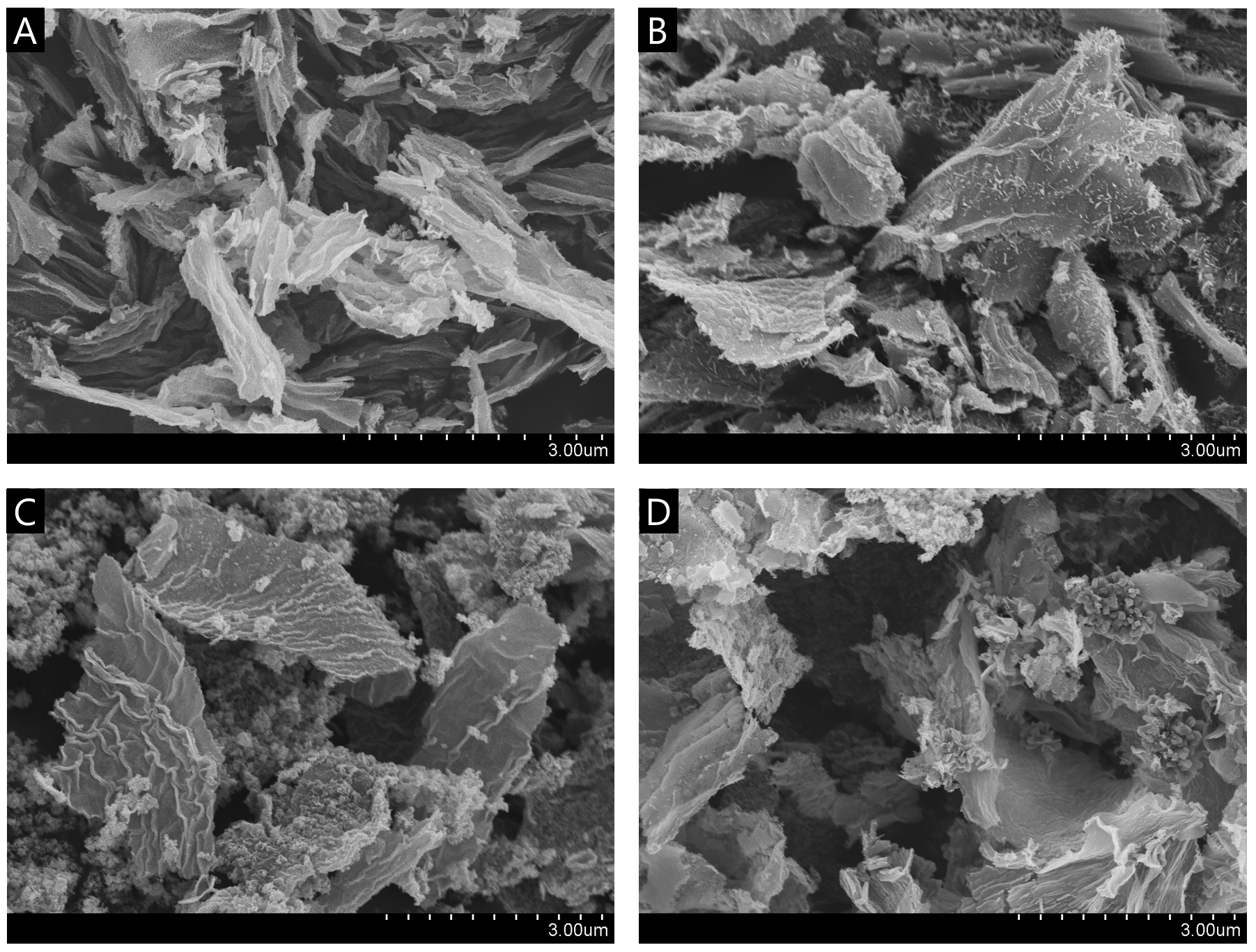


Figure S1. (A-D) SEM images of RuO_2_, FRO, BRO and BFRO, respectively.


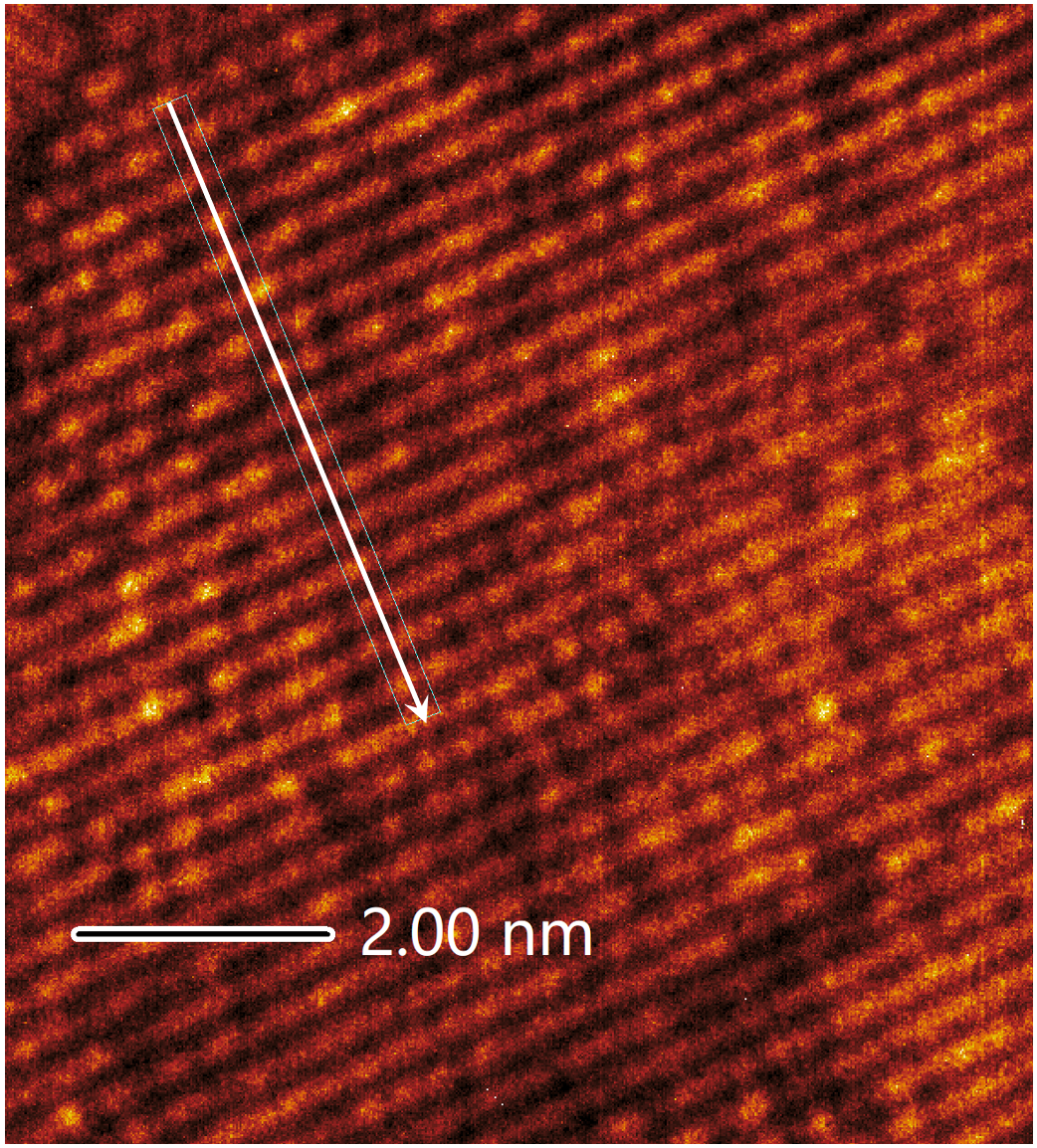


Figure S2. Lattice fringes of BFRO. The fringe spacing is estimated to be 3.118 Å, which is consistent with the interplanar distance of the (110) crystal facet of RuO_2_.


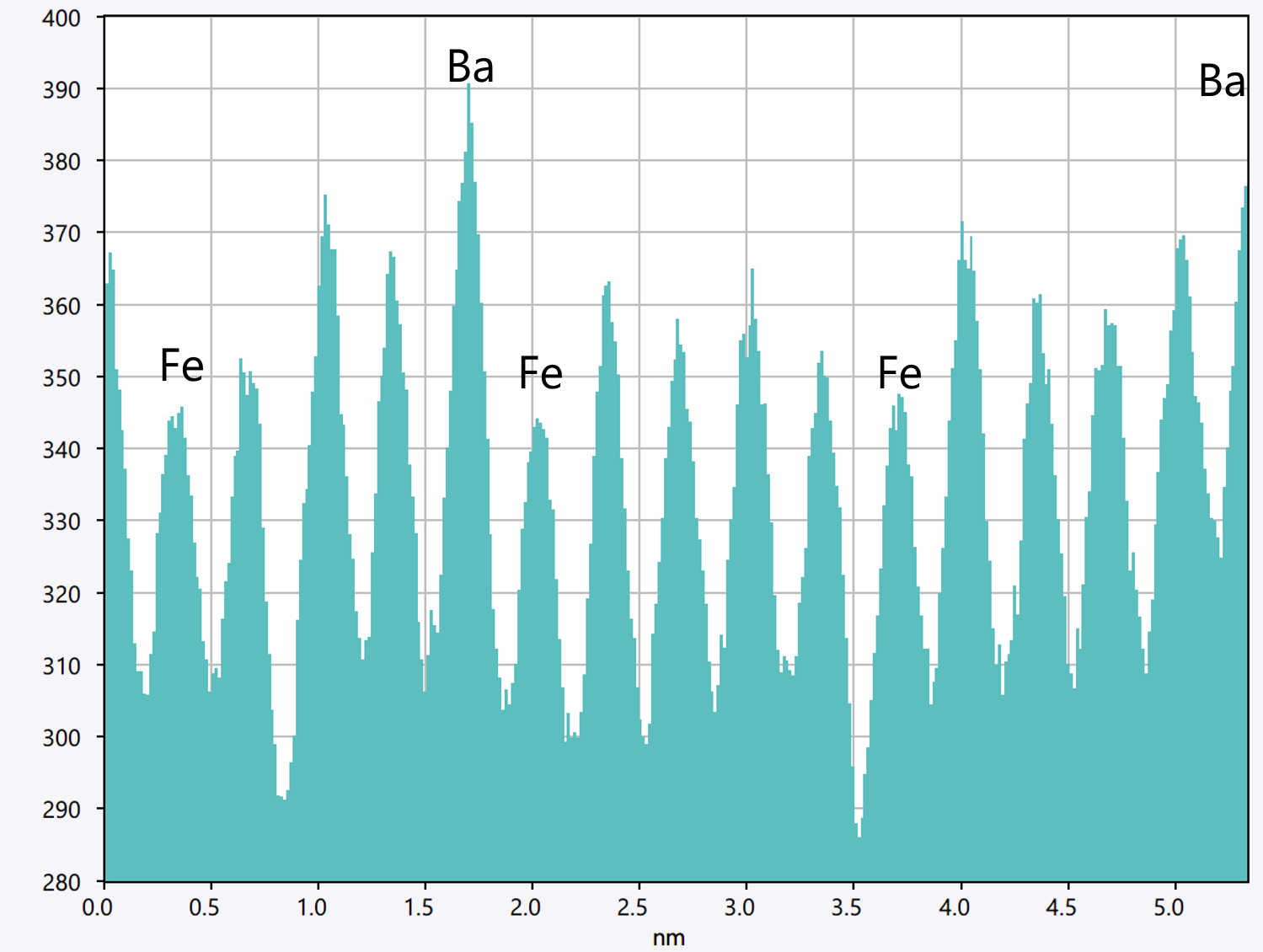


Figure S3. Brightness distribution along the white arrow in Figure S2. Three distinct brightness levels can be identified, corresponding respectively to the highest intensity of Ba atoms, the intermediate intensity of Ru atoms, and the lowest intensity of Fe atoms.


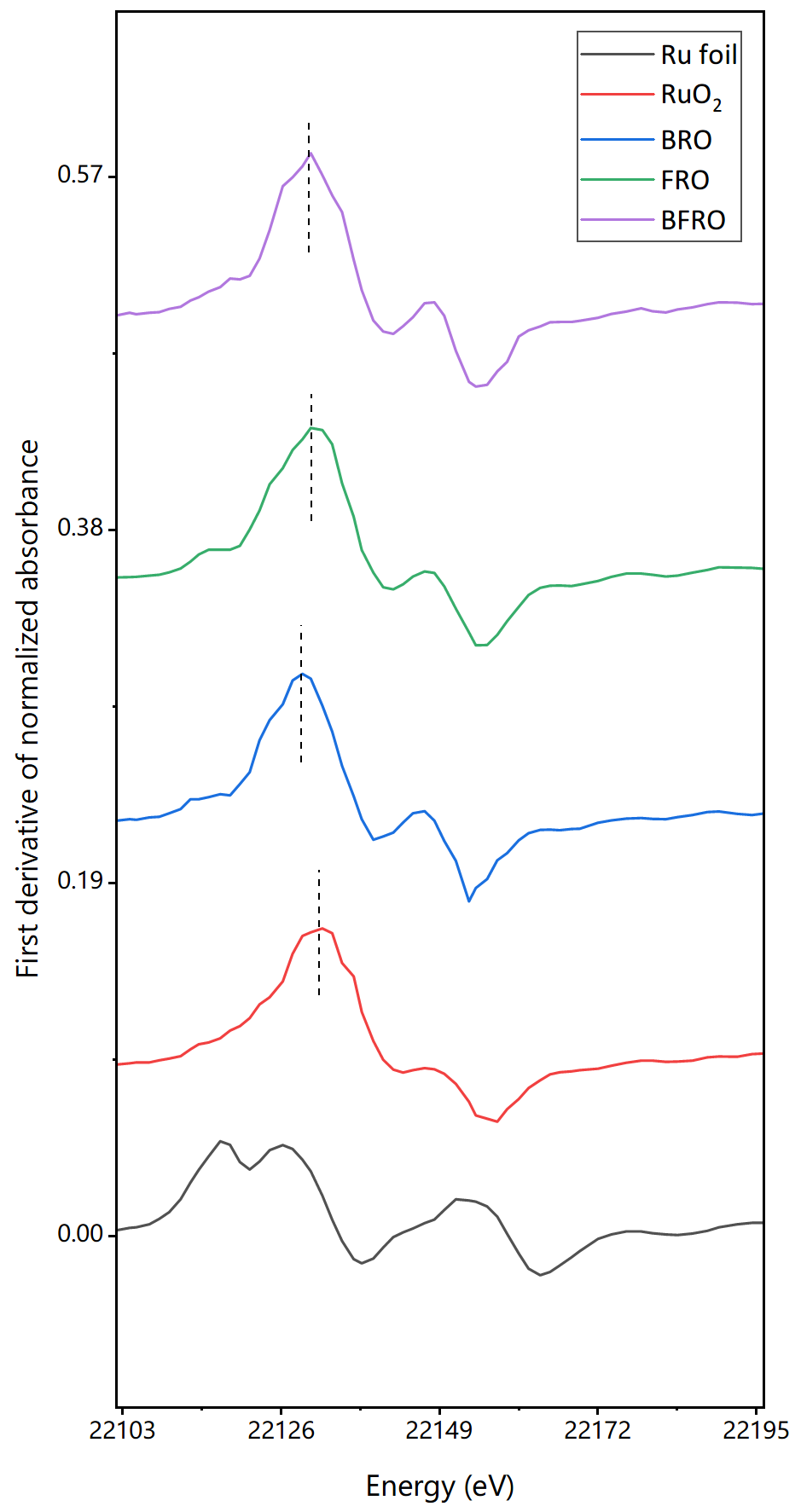


Figure S4. First derivative of normalized XANES data.


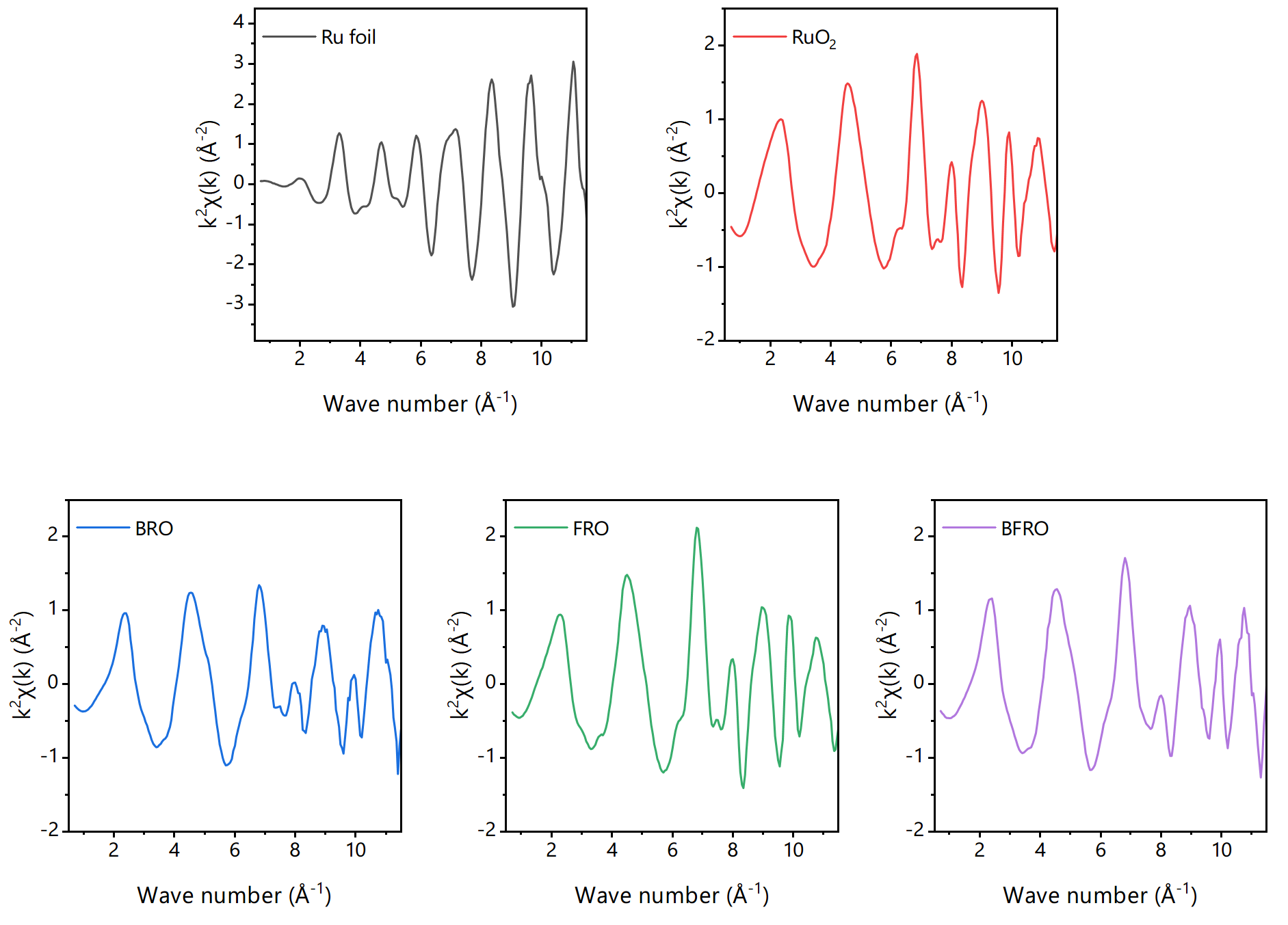


Figure S5. EXAFS data in k-space.


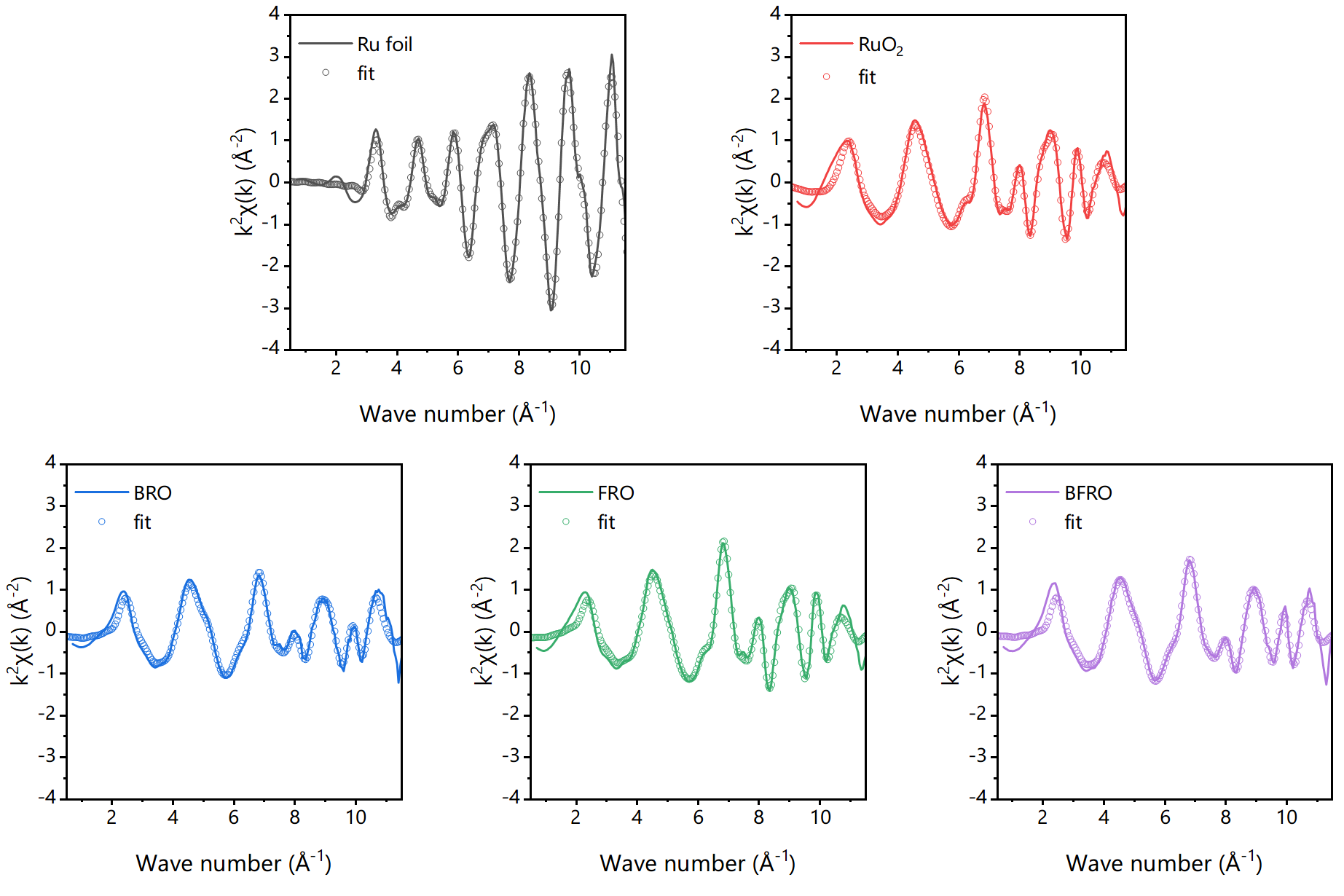


Figure S6. Fitting of EXAFS data in k-space.


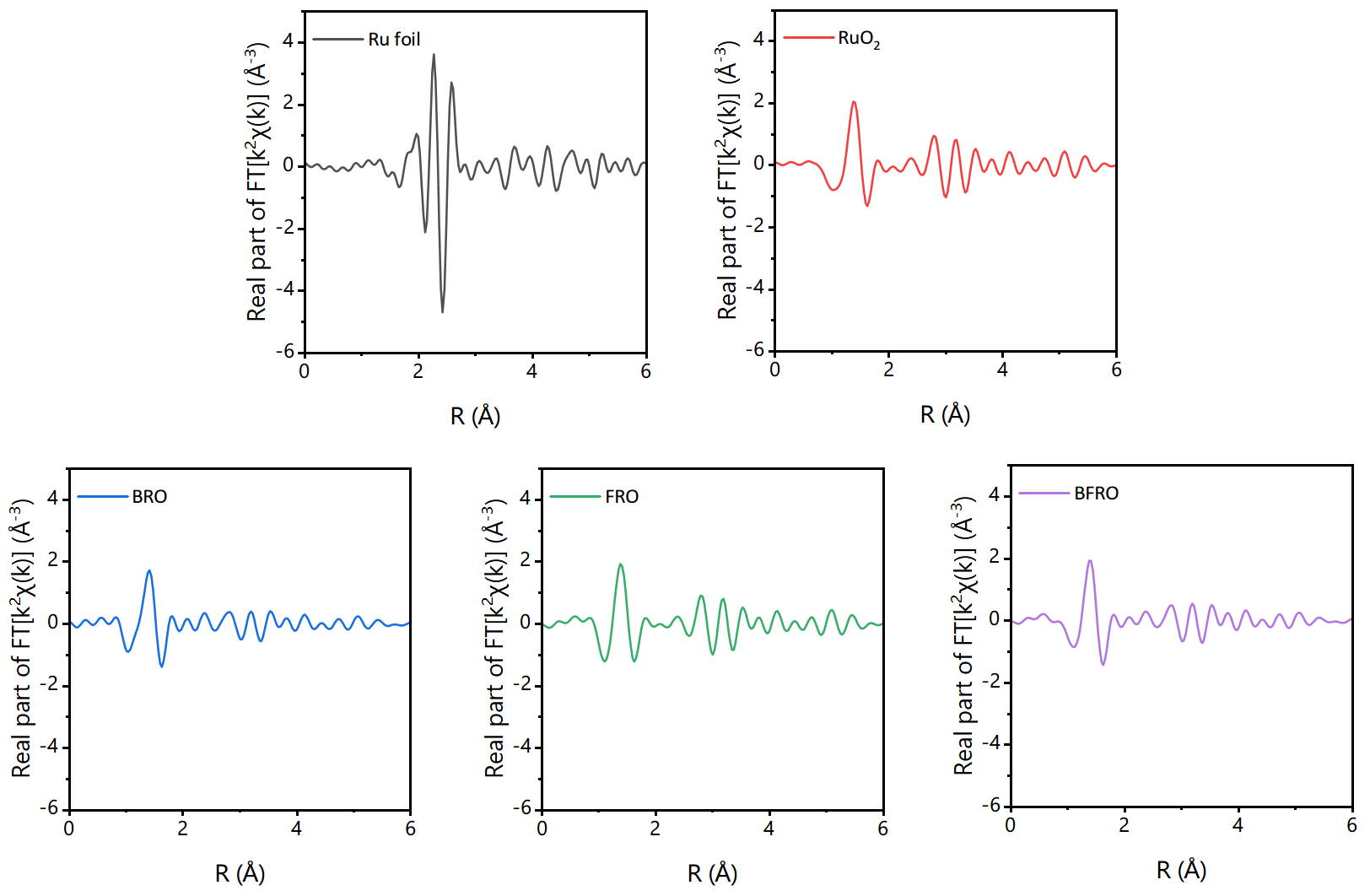


Figure S7. Real part of FT [k^2^χ(k)].


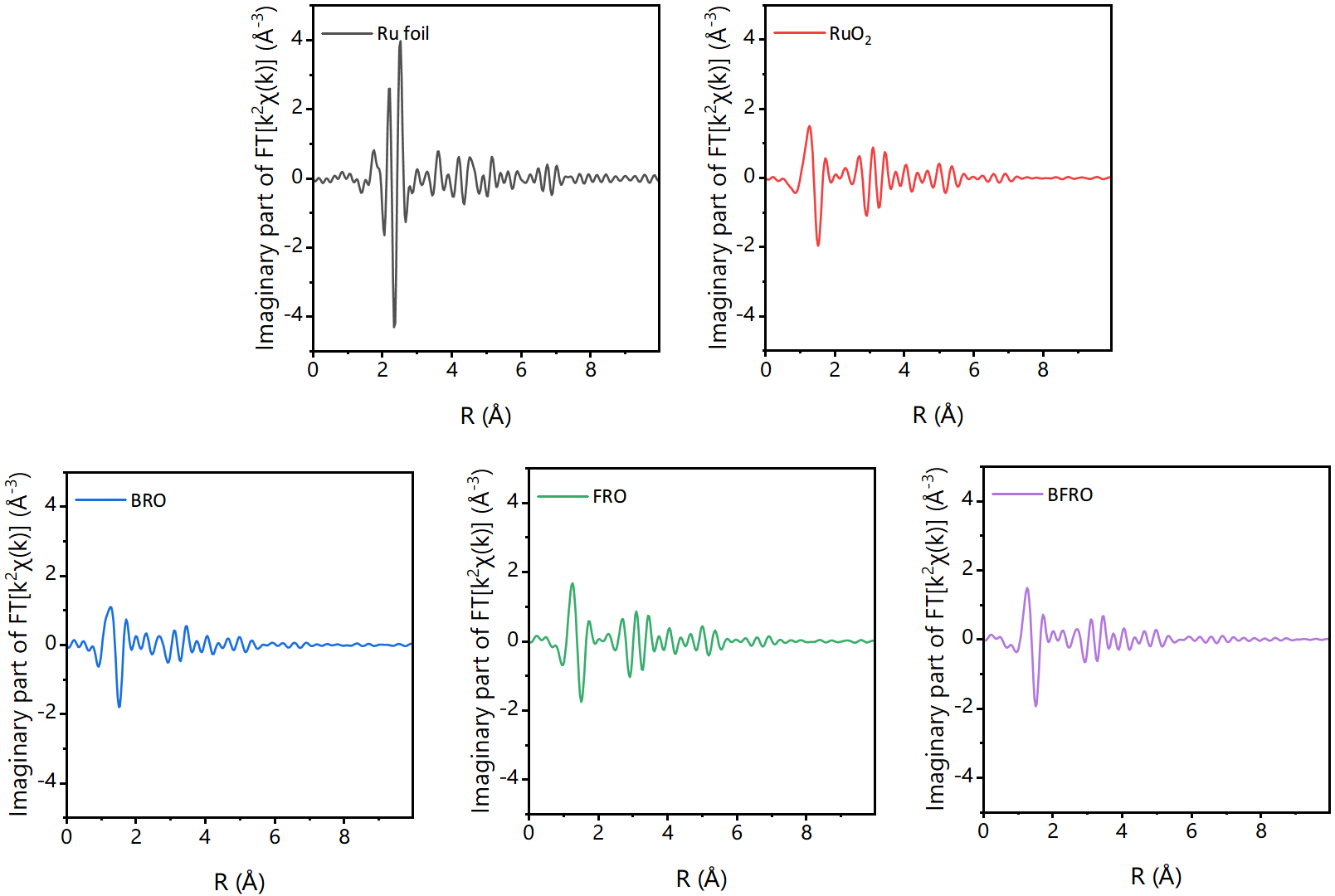


Figure S8. Imaginary part of FT [k2χ(k)].


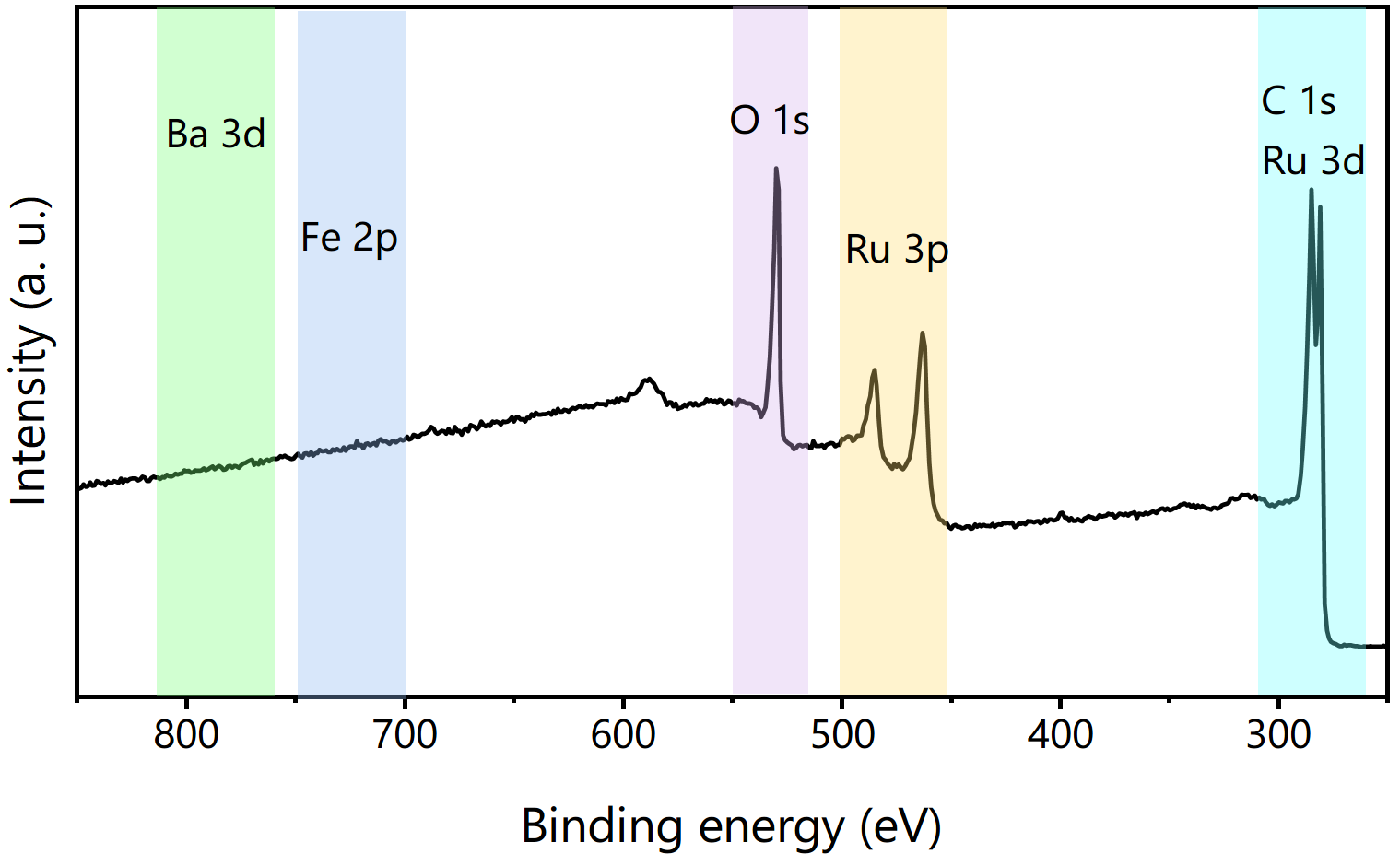


Figure S9. Full XPS survey of RuO_2_.


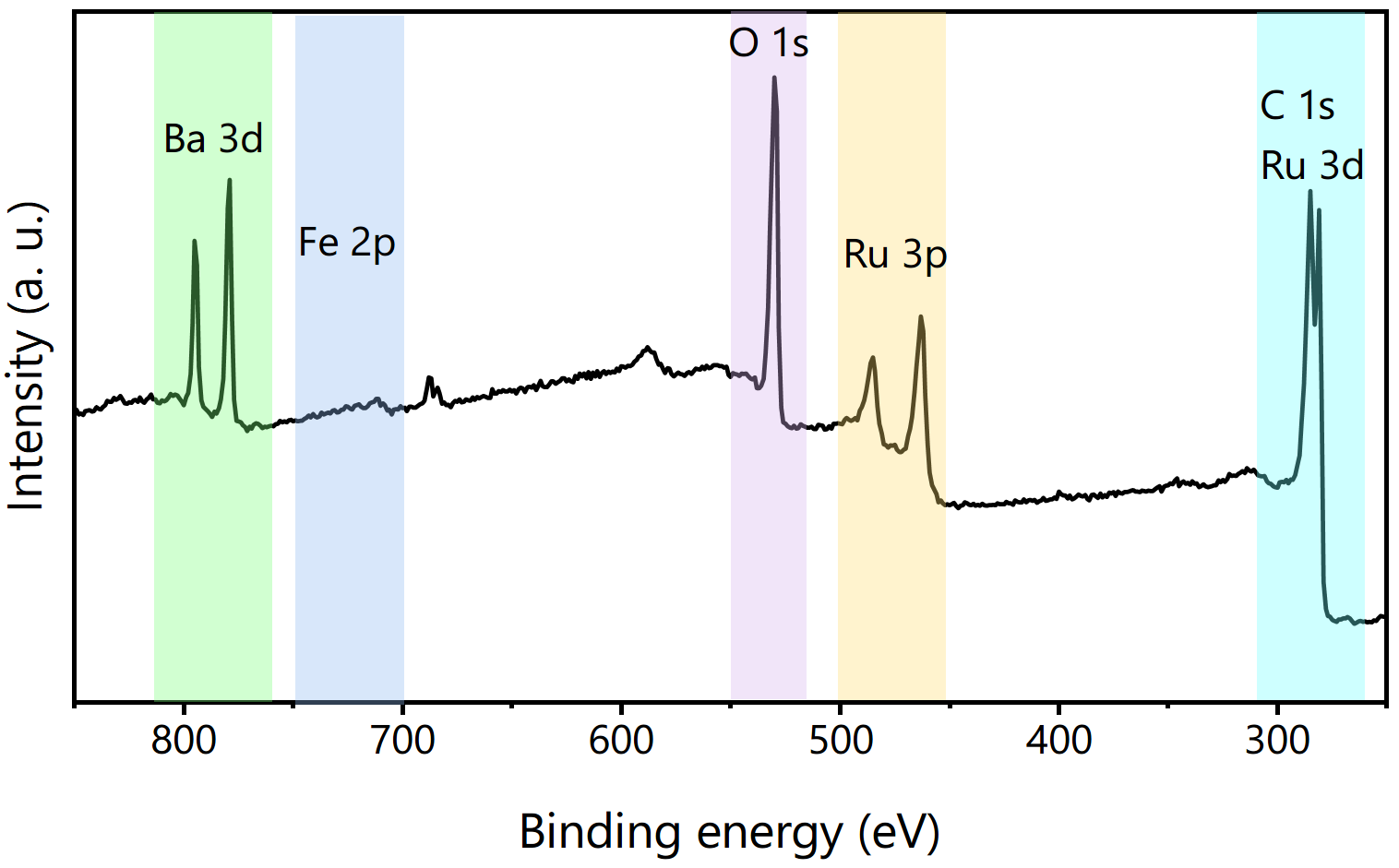


Figure S10. Full XPS survey of BRO.


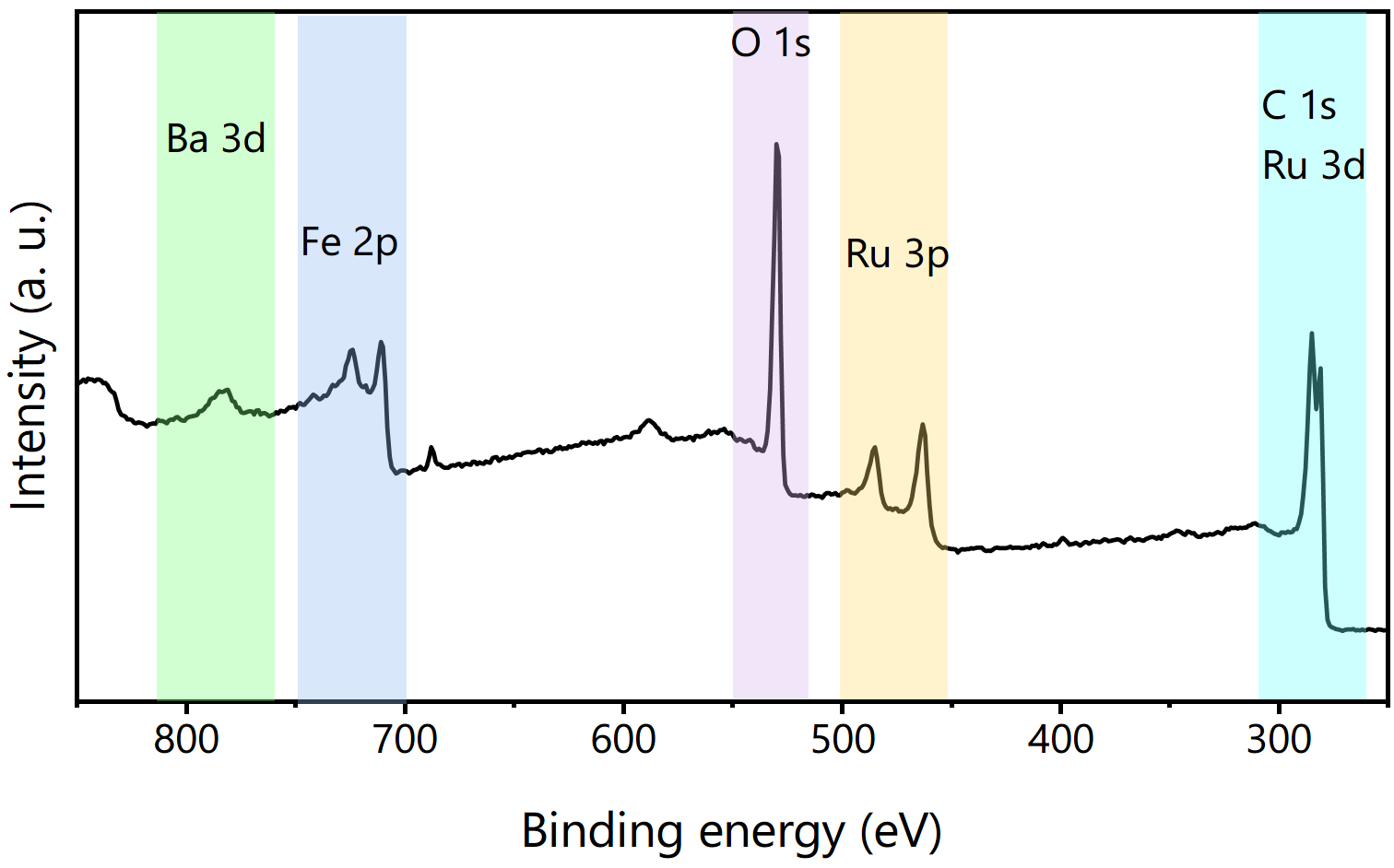


Figure S11. Full XPS survey of FRO.


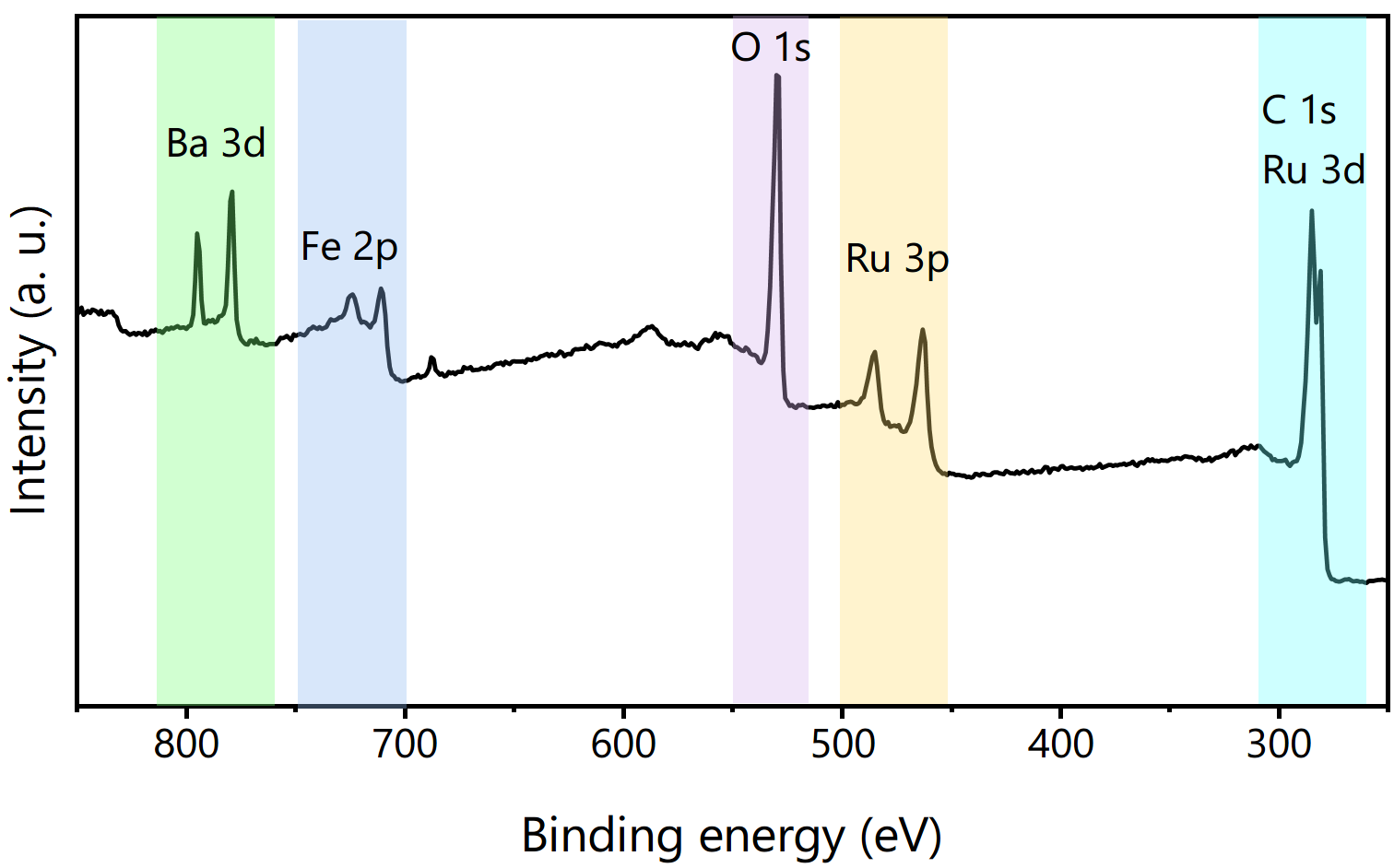


Figure S12. Full XPS survey of BFRO.


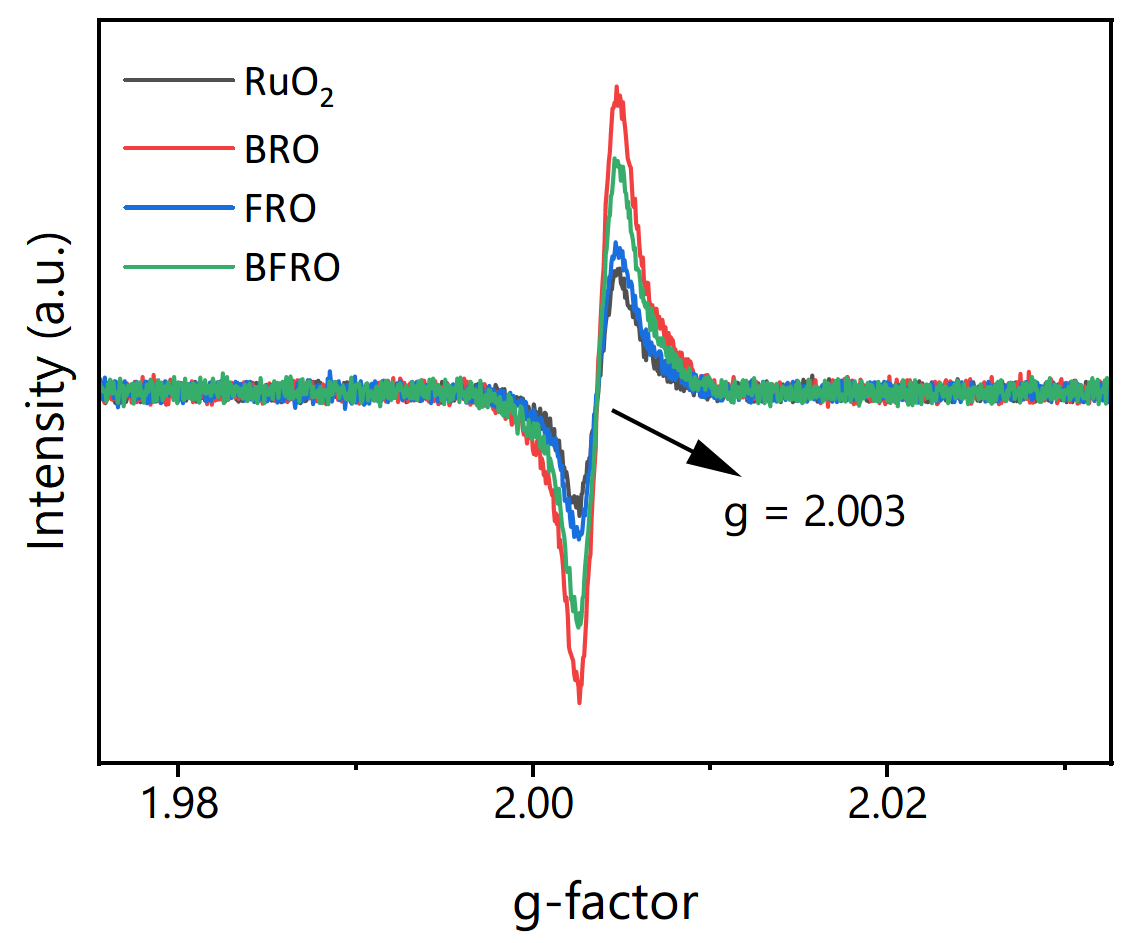


Figure S13. EPR measurements.

Table S3. Summary of the overpotentials and Tafel slopes of the corresponding samples measured in acidic, alkaline, and neutral electrolytes.

|  | Acid electrolyte | | | | Alkaline electrolyte | | | | Neutral electrolyte | | | |
| --- | --- | --- | --- | --- | --- | --- | --- | --- | --- | --- | --- | --- |
| Sample | RuO_2_ | BFO | FRO | BFRO | RuO_2_ | BFO | FRO | BFRO | RuO_2_ | BFO | FRO | BFRO |
| η_10_  (mV) | 297 | 237 | 241 | 174 | 331 | 268 | 286 | 236 | 425 | 325 | 356 | 284 |
| Tafel slope  mV/dec | 94.4 | 69.9 | 74.7 | 58.5 | 112.7 | 63.9 | 86.2 | 60 | 191 | 128.8 | 183 | 104.4 |

η_10_: overpotential at a current density of 10 mA/cm^2^.


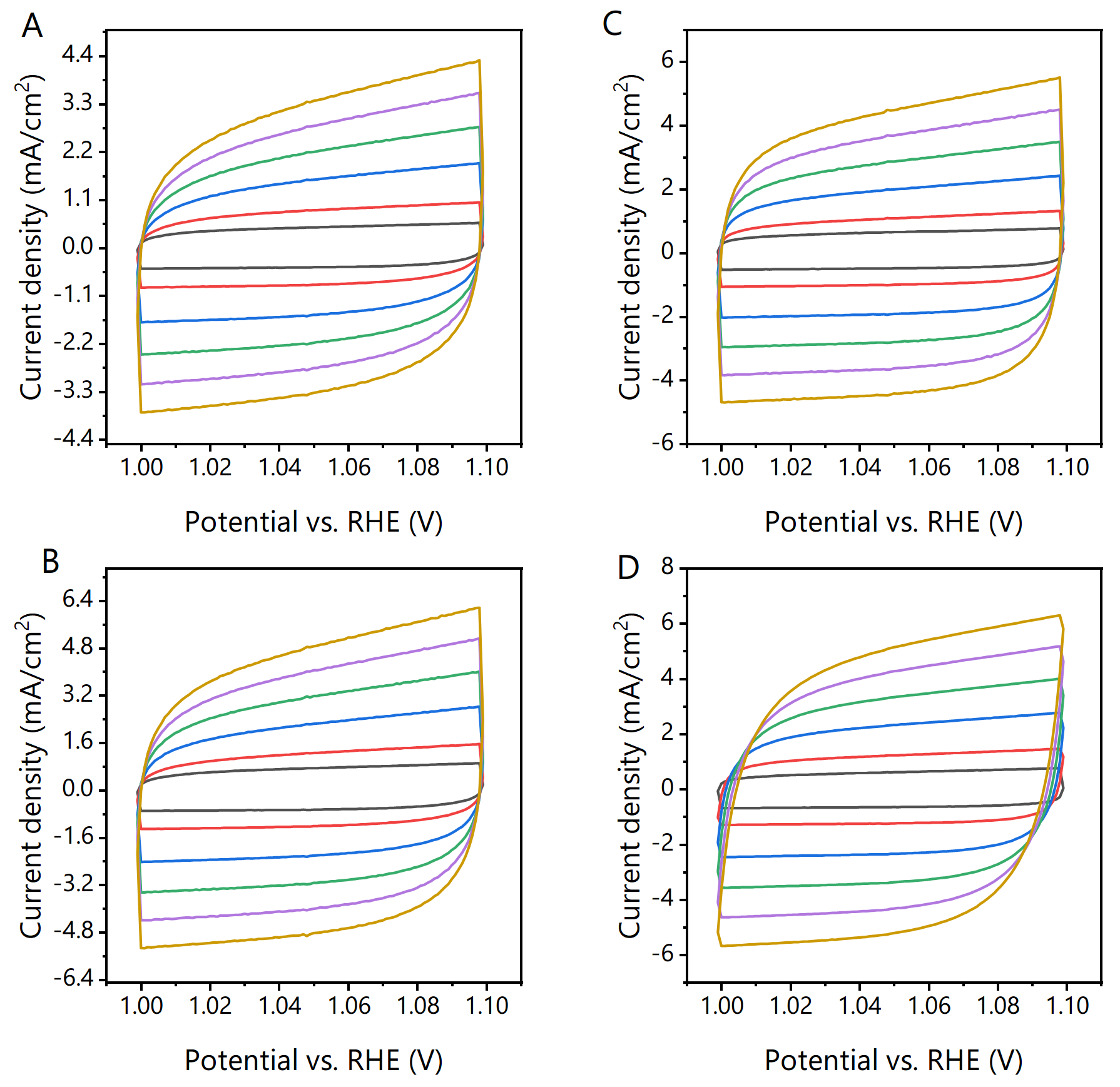


Figure S14. Circulation voltammetry sweeps of corresponding samples (A for RuO_2_, B for BRO, C for FRO, D for BFRO) in acid electrolyte.


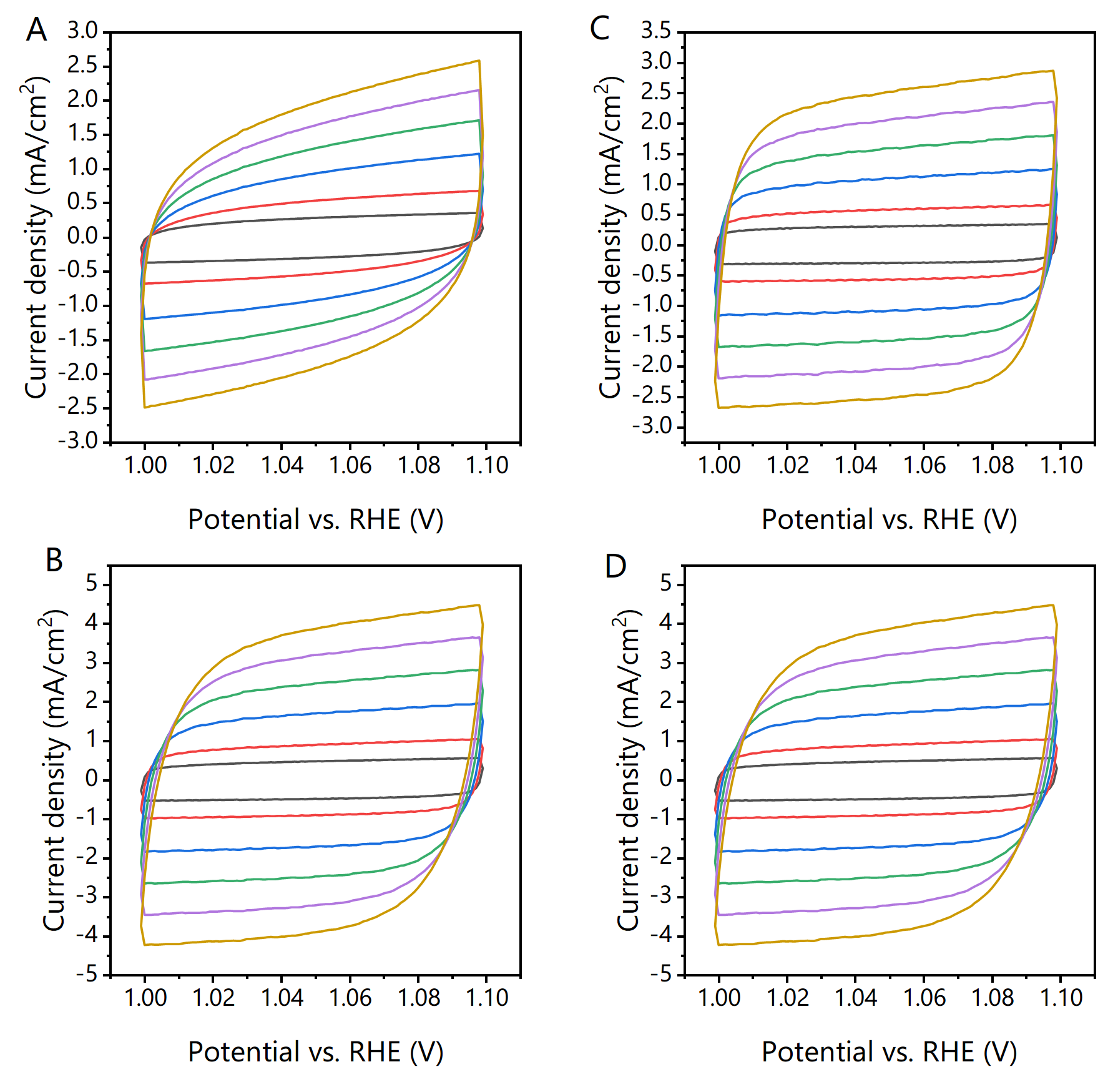


Figure S15. Circulation voltammetry sweeps of corresponding samples (A for RuO_2_, B for BRO, C for FRO, D for BFRO) in alkaline electrolyte.


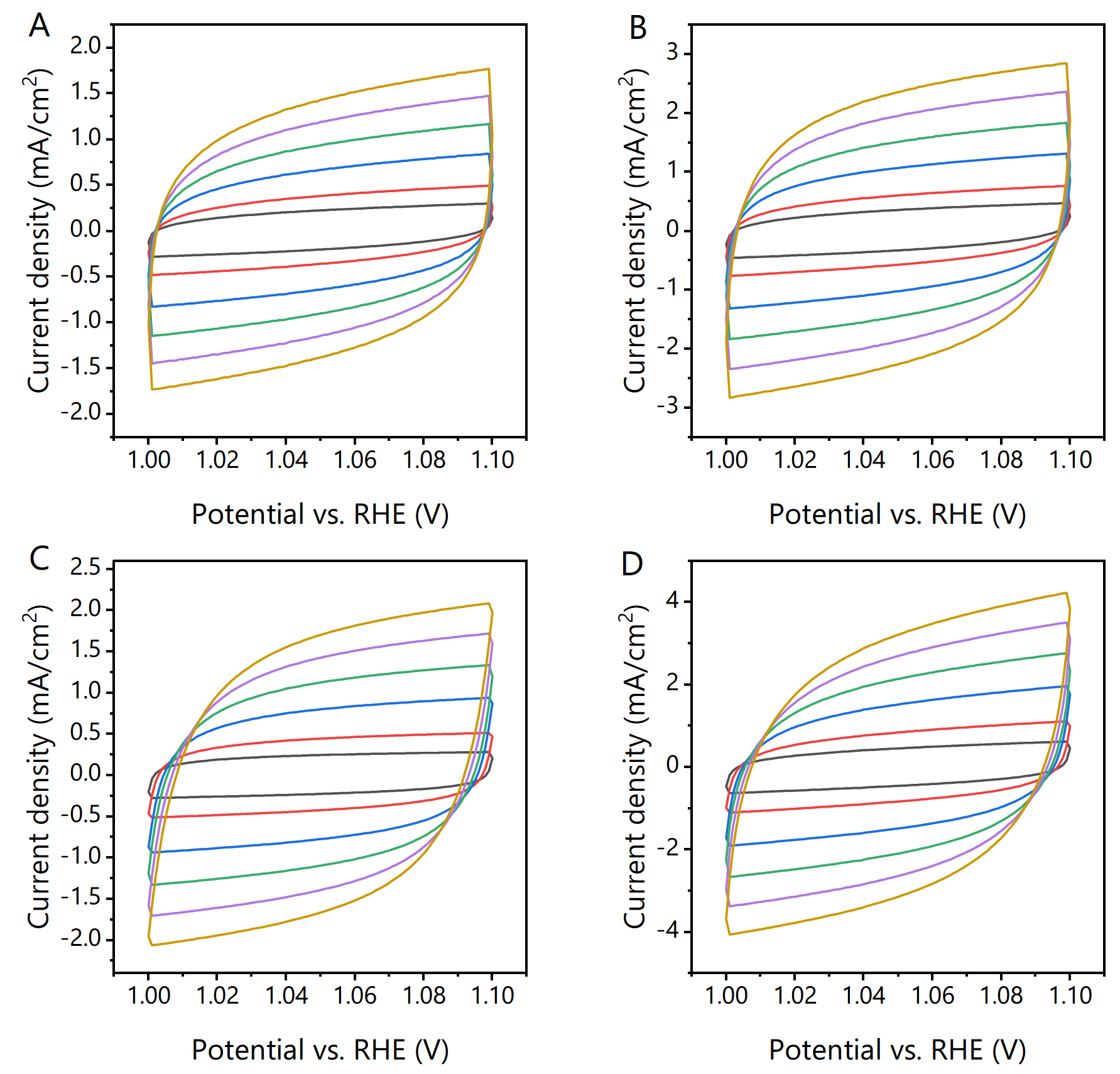


Figure S16. Circulation voltammetry sweeps of corresponding samples (A for RuO_2_, B for BRO, C for FRO, D for BFRO) in neutral electrolyte.

Table S4. C_dl_, ECSA and EIS of samples in various electrolytes.

|  | **Sample** | **C_dl_**  (mF cm^-2^) | **ECSA**  (cm^2^) | **EIS** | |
| --- | --- | --- | --- | --- | --- |
|  |  |  |  | **R_s_** (Ω) | **R_ct_** (Ω) |
| acid  electrolyte | RuO_2_ | 16.0 | 267 | 6.3 | 205.4 |
|  | BRO | 23.0 | 384 | 7.49 | 97 |
|  | FRO | 21.7 | 361 | 4.93 | 139.9 |
|  | BFRO | 25.2 | 420 | 8.05 | 74.44 |
| alkaline  electrolyte | RuO_2_ | 9.1 | 228 | 12.76 | 243.7 |
|  | BRO | 15.9 | 398 | 10.47 | 126.3 |
|  | FRO | 12.3 | 308 | 9.6 | 213.7 |
|  | BFRO | 18.9 | 473 | 10.4 | 102.9 |
| neutral  electrolyte | RuO_2_ | 6.6 | 166 | 33.92 | 455.6 |
|  | BRO | 10.9 | 272 | 32.73 | 157.1 |
|  | FRO | 8.0 | 200 | 34.72 | 265 |
|  | BFRO | 15.1 | 378 | 31.43 | 142.2 |


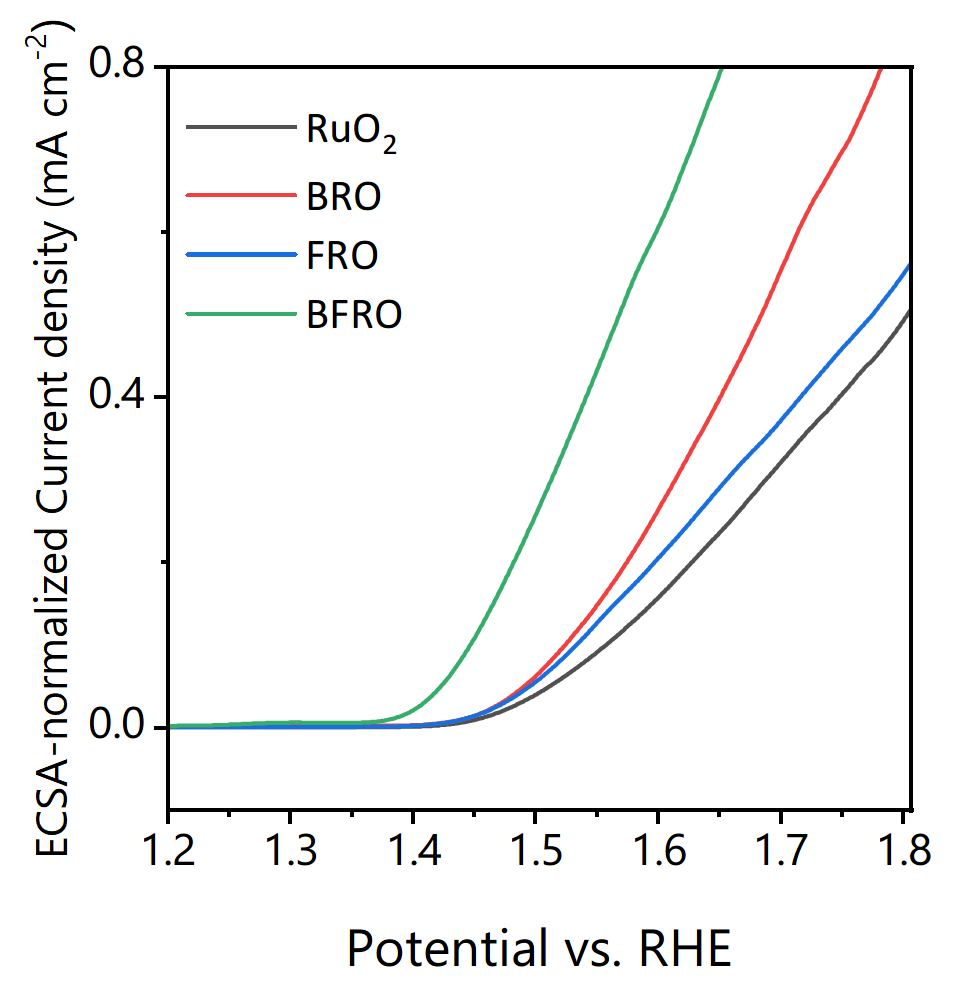


Figure S17. LSV curves normalized by ECSA in acid electrolyte.


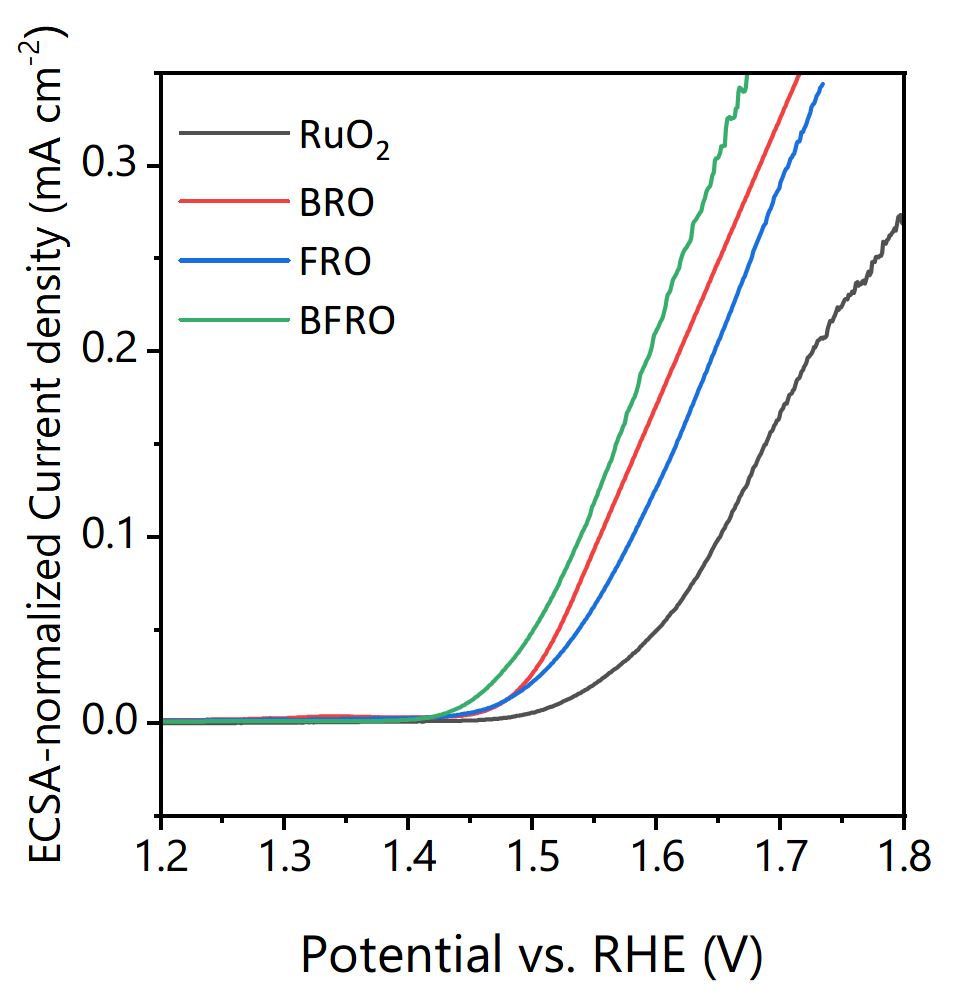


Figure S18. LSV curves normalized by ECSA in alkaline electrolyte.


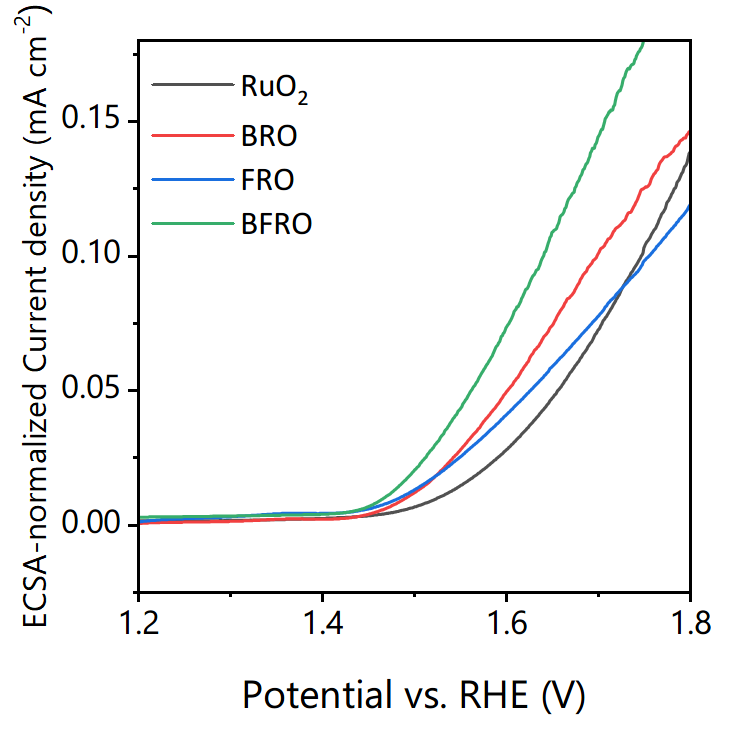


Figure S19. LSV curves normalized by ECSA in neutral electrolyte.

Table S5. Comparison of the OER performance of RuO_2_-based catalysts.

| Catalysts | Electrolytes | η_10_  (mV) | Reference |
| --- | --- | --- | --- |
| IrRu HNWs | 0.5 M H_2_SO_4_ | 215 | Joule 2024, 8, 450 |
| Ru-VO_2_ | 0.5 M H_2_SO_4_ | 228 | Adv. Mater. 2024, 36, 2310690 |
| MD-RuO_2_-BN | 0.5 M H_2_SO_4_ | 196 | Nat. Commun. 2024, 15, 3982 |
| RuIrFeCoNiO_2_ | 0.5 M H_2_SO_4_ | 210 | Sci. Adv. 2023, 9, eadf9144 |
| SS Pt-RuO_2_ HNSs | 0.5 M H_2_SO_4_ | 228 | Sci. Adv. 2022, 8, eabl9271 |
| Ni-RuO_2_ | 0.5 M H_2_SO_4_ | 214 | Nat. Mater. 2023, 22, 100 |
| Rh-RuO_2_-G | 0.5 M H_2_SO_4_ | 161 | Nat. Commun. 2023, 14, 1412 |
| Lix-RuO_2_ | 0.5 M H_2_SO_4_ | 156 | Nat. Commun. 2022, 13, 3784 |
| Ru(anc)-Co_3_O_4_ | 0.5 M H_2_SO_4_ | 198 | J. Am. Chem. Soc. 2023, 145, 23659 |
| Ru@V-RuO_2_/C | 0.5 M H_2_SO_4_ | 176 | Adv. Mater. 2023, 35, 2206351 |
| Ru/Co-N-C | 0.5 M H_2_SO_4_ | 232 | Adv. Mater. 2022, 34, 2110103 |
| RuMn | 0.5 M H_2_SO_4_ | 240 | Adv. Funct. Mater. 2022, 32, 2200131 |
| Ru/ZnRuO_2_ | 0.5 M H_2_SO_4_ | 184 | Adv. Funct. Mater., 2024, 34, 2409306. |
| In–RuO_2_/G | 0.5 M H_2_SO_4_ | 187 | Angew. Chem. Int. Ed. 2024, 63, e202316903 |
| Ru@V-RuO_2_/C | 0.5 M H_2_SO_4_ | 176 | Adv. Mater. 2023, 35, 2206351 |
| Nd_0.1_RuO_x_ | 0.5 M H_2_SO_4_ | 211 | Adv. Funct. Mater. 2023, 33, 2213304 |
| C-RuO_2_-RuSe | 0.5 M H_2_SO_4_ | 212 | Chem 2022, 8(6), 1673 |
| Mn–RuO_2_ | 0.5 M H_2_SO_4_ | 158 | ACS Catal. 2020, 10(2), 1152 |
| Mo–RuO_2_ | 0.5 M H_2_SO_4_ | 147 | Small 2024, 20, 2305889 |
| Ru_0.85_Zn_0.15_O_2−δ_ | 0.5 M H_2_SO_4_ | 190 | Adv. Energy Mater. 2023, 13, 2300177 |
| Cu–RuO_2_ | 0.5 M H_2_SO_4_ | 188 | Adv. Mater. 2018, 1801351 |
| a/c–RuO_2_/CC | 0.5 M H_2_SO_4_ | 150 | Mater. Horiz. 2023, 10, 2904 |
| Y_2_Ru_2_O_7−δ_ | 0.1 M HClO_4_ | 270 | J. Am. Chem. Soc. 2017, 139, 12076 |
| Co–RuO_2_ | 0.5 M H_2_SO_4_ | 328 | ACS Appl. Mater. Interfaces 2022, 14(1), 1077 |
| Cr0.6Ru_0.4_O_2_ | 0.5 M H_2_SO_4_ | 178 | Nat. Commun. 2019, 10, 162 |
| RuIr@CoNC | 0.5 M H_2_SO_4_ | 223 | ACS Catal. 2021, 11(6), 3402 |
| IrxRu1−xO2 | 0.5 M H_2_SO_4_ | 211 | Nano Research 2022, 488 |
| RuO2–WC NPs | 0.5 M H_2_SO_4_ | 347 | Angew. Chem. Int. Ed. 2022, e202202519 |
| IrNiCu double-layered nanoframe | 0.1 M HClO_4_ | 300 | ACS Nano, 2017,11,5500. |
| IrO_2_-RuO_2_@Ru | 0.5 M H_2_SO_4_ | 281 | J. Mater. Chem. A, 2017,5, 17221 |
| MCN–RuO₂ | 1.0 M KOH | 200 | Small Sci., 2025, 0, e202500546. |
| HN-Ru/RuO₂ | 1.0 M KOH | 295 | Nanoscale Adv. 2024, 6, 867–875. |
| RuO₂/TiO₂ | 1.0 M KOH | 260 | J. Mater. Sci. 2024, 59, 10193–10206. |
| A-RRO@G | 1.0 M KOH | 222.4 | J. Mater. Chem. A 2025, 13, 5091–5105. |
| Ru@V–RuO_2_/C HMS | 1.0 M KOH | 201 | Adv. Mater. 2023, 35, 2206351 |
| Ru/RuO_2_ (Ru-30) | 1.0 M KOH | 200 | Appl. Catal. B 2022, 307, 121199 |
| Ru_0.85_Zn_0.15_O_2−δ_ | 1.0 M KOH | 195 | Adv. Energy Mater. 2023, 13, 2300177 |
| Ru NCs/P,O–NiFe LDH/NF | 1.0 M KOH | 175 | Adv. Funct. Mater. 2024, 34, 2310690 |
| Ru–RuO_2_@NPC | 1.0 M KOH | 190 | Appl. Catal. B 2022, 302, 120838 |
| Ni/RuOx@C | 1.0 M KOH | 250 | Chem. Eng. J. 2021, 426, 130762 |
| RuO_2_–250 | 1.0 M KOH |  | Chem. Eng. J. 2021, 425, 131707 |
| Ni–Ru NWs | 1.0 M KOH | 194 | Energy Environ. Sci. 2021, 14, 3194 |
| Pd/NiFeOx | 1.0 M KOH | 180 | Adv. Funct. Mater. 2021, 31, 2107181 |
| RuO₂–Fe₂O₃ | 1.0 M KOH | 290 | Int. J. Hydrogen Energy 2023, 48, 1813–1830. |
| RuO₂/Co₃O₄ | 1.0 M KOH | 302 | Int. J. Hydrogen Energy 2020, 45, 9575–9582. |
| RuO₂/CeO₂ | 1.0 M KOH | 350 | Int. J. Hydrogen Energy 2020, 45, 18635–18644. |
| Ni₁.₂₅Ru₀.₇₅P | 1.0 M KOH | 340 | Chem. Mater. 2022, 34, 6255–6267. |
| CoOₓ/RuO₂ | 1.0 M KOH | 230 | Appl. Surf. Sci. 2022, 589, 152958. |
| Co-SAC/RuO₂ | 1.0 M KOH | 200 | Angew. Chem. Int. Ed. 2022, 61, e202114951. |
| RuCu | 1.0 M KOH | 234 | Angew. Chem. Int. Ed. 2019, 58, 13983–13988. |
| RuO₂ NWs | 1.0 M KOH | 224 | Adv. Funct. Mater. 2018, 28, 1803722. |
| Ru-VO_2_ | 1 M PBS | 269 | Adv. Mater. 2024, 36, 2310690. |
| Ir@Ni-NDC | 1 M PBS | 296 | Angew. Chem. Int. Ed. 2023, 62, 202302220 |
| IrO_2_/V_2_O_5_ | 1 M PBS | 329 | Adv. Sci. 2022, 9, 2104636 |
| Ir-NR/C | 1 M PBS | 305 | Appl. Catal. B 2020, 279, 119394 |
| IrW nanobranches | 1 M PBS | 326 | Nanoscale 2019, 11, 8898 |
| Li–IrSe_2_ | 1 M PBS | 270 | Angew. Chem. Int. Ed. 2019, 58, 14764–14769 |
| a/c RuO_2_ | 1 M PBS | 235 | Angew. Chem. Int. Ed. 2021, 60, 18821–18829 |
| Ir-NSG | 1 M PBS | 297 | Nat. Commun. 2020, 11, 4246 |
| IrO2–RuO_2_/C | PBS | 290 | Energy Fuels 2022, 36, 1015 |
| BFRO | 0.5 M H_2_SO_4_ | 174 | This work |
|  | 1.0 M KOH | 236 |  |
|  | 1 M PBS | 284 |  |


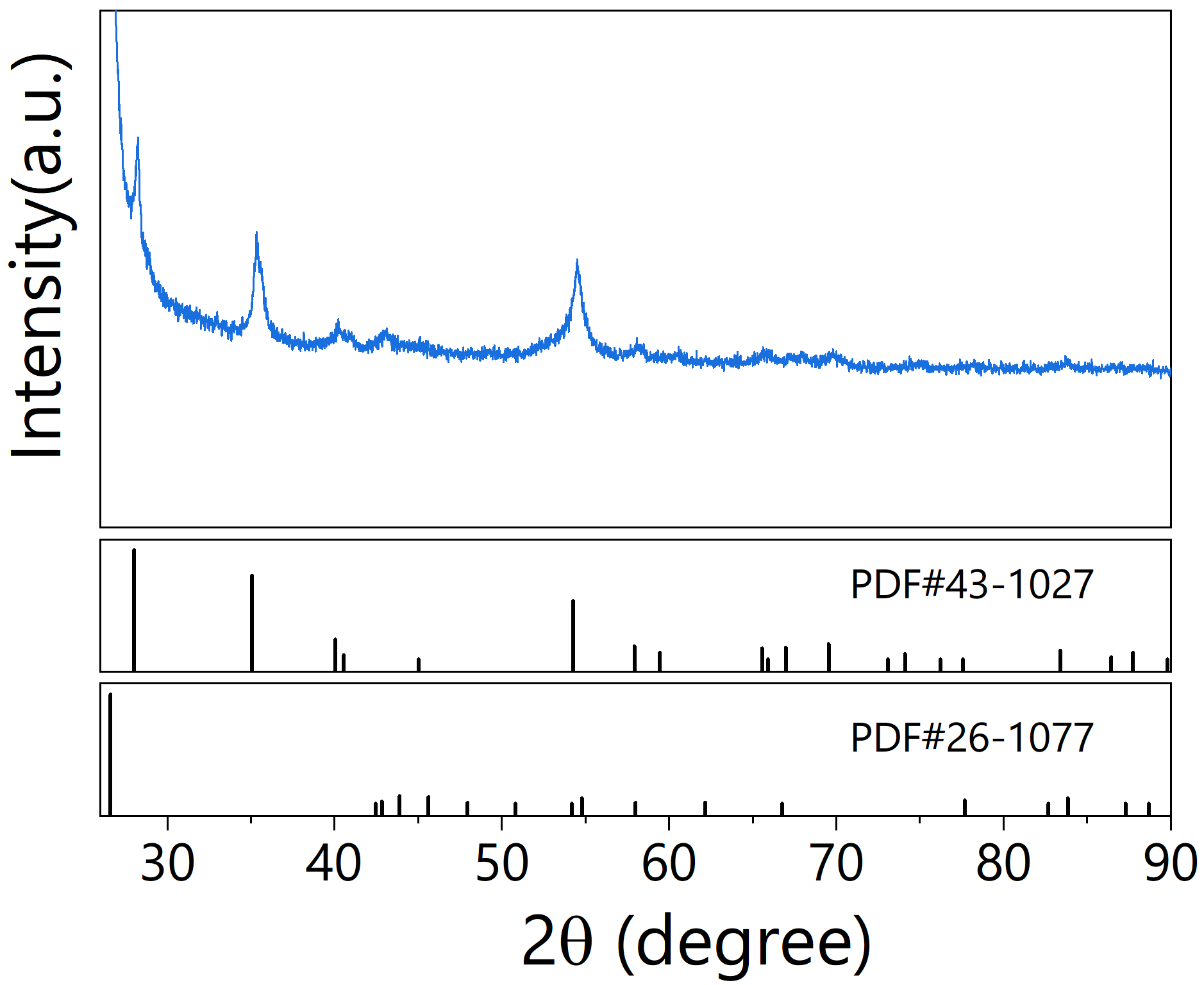


Figure S20. XRD patten of BFRO after stability test.


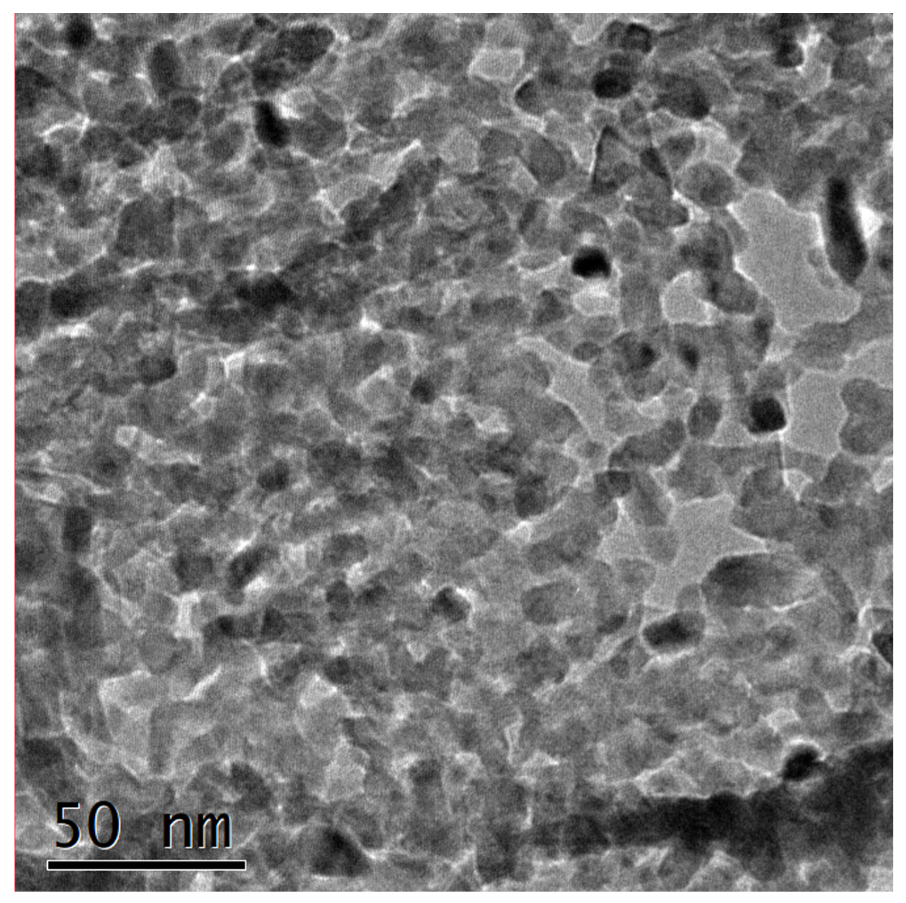


Figure S21. TEM image of BFRO after stability test.


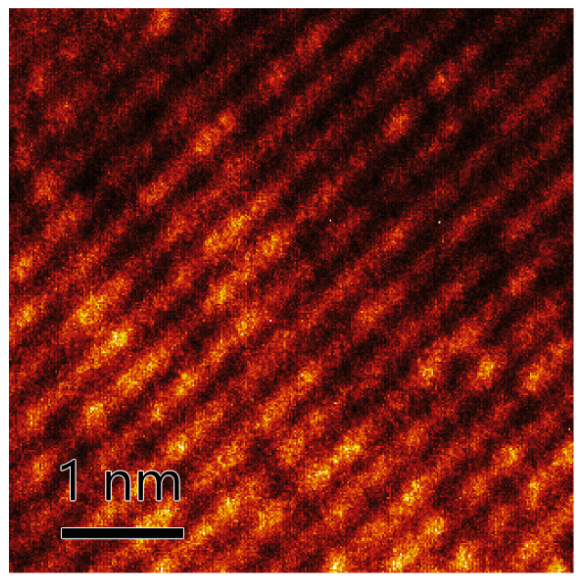


Figure S22. Atom-resolved TEM image of BFRO after stability test.


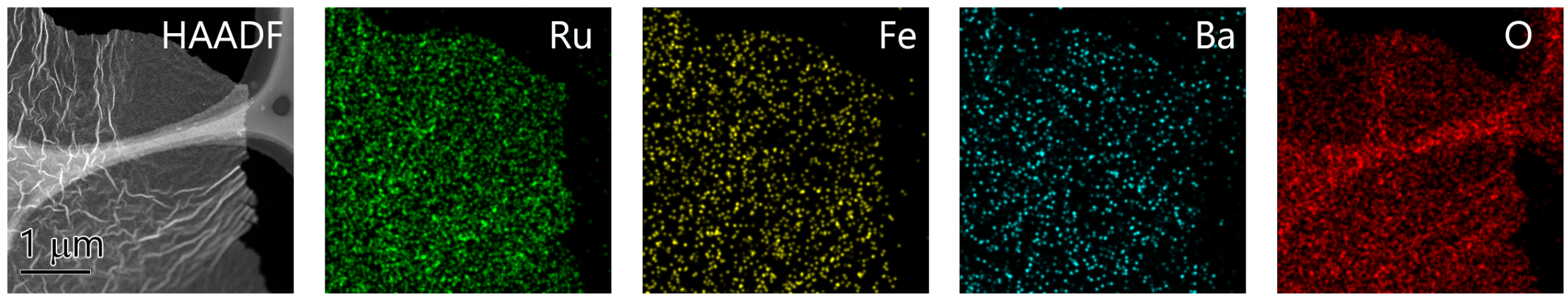


Figure S23. Element mapping of BFRO after stability test.


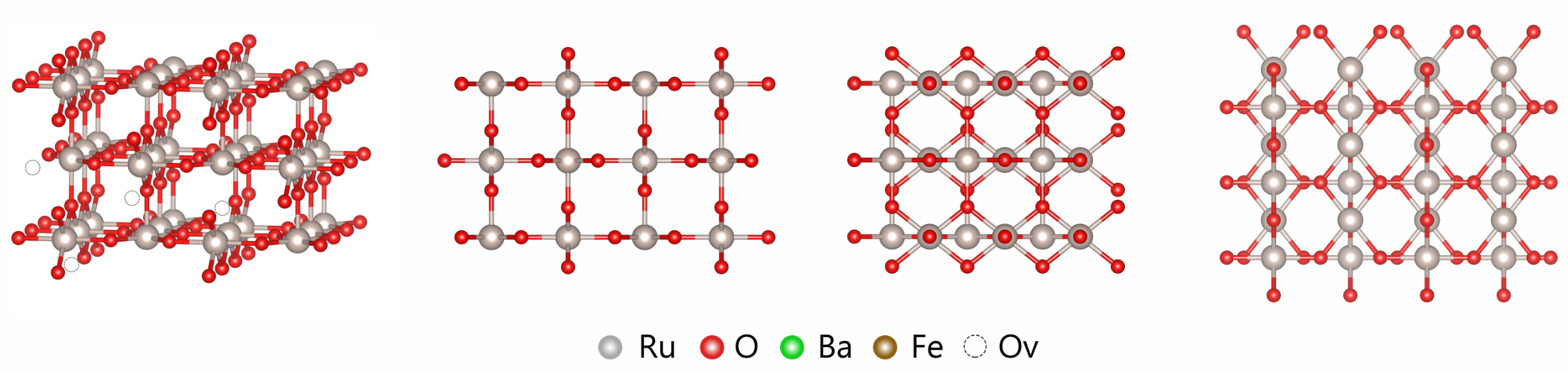


Figure S24. DFT calculation model of RuO_2_.


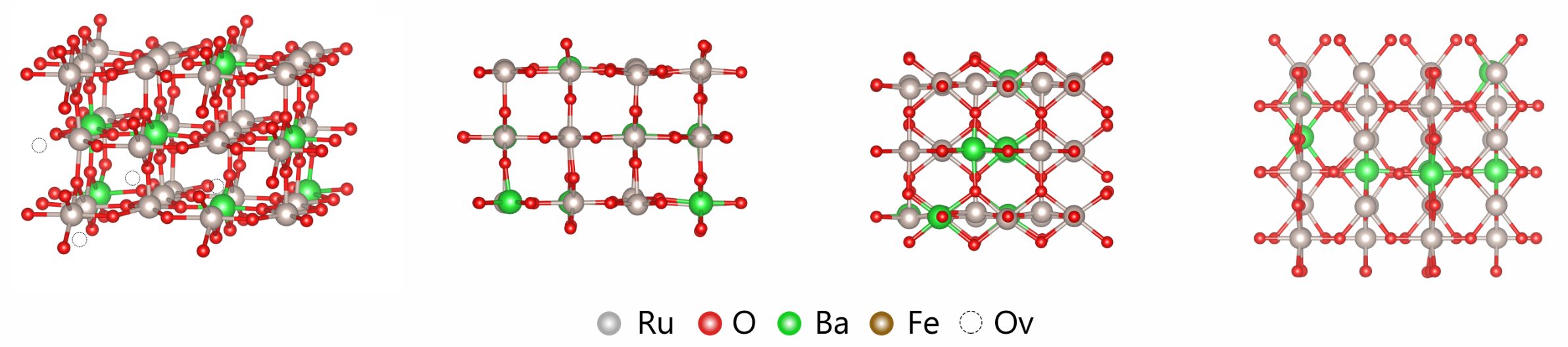


Figure S25. DFT calculation model of BFO.


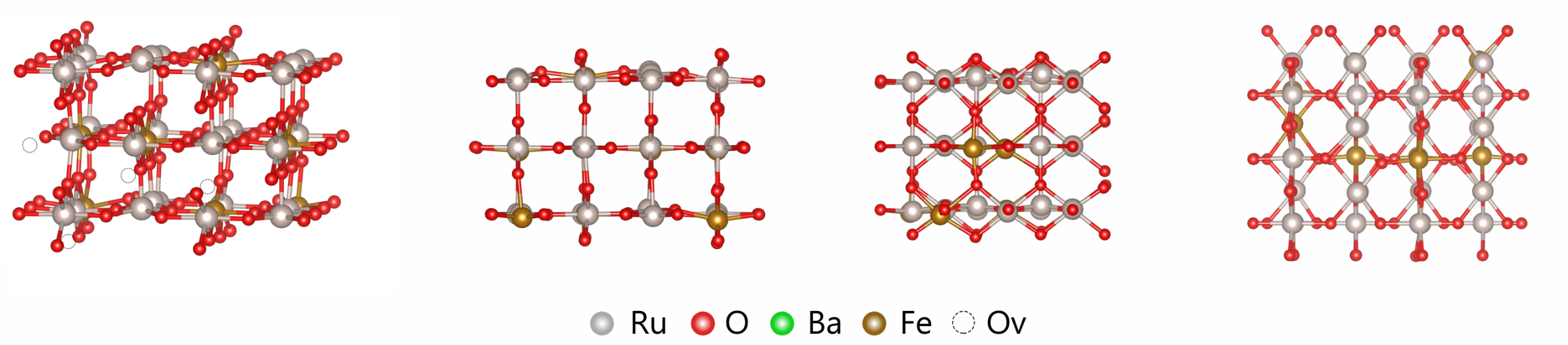


Figure S26. DFT calculation model of FRO.


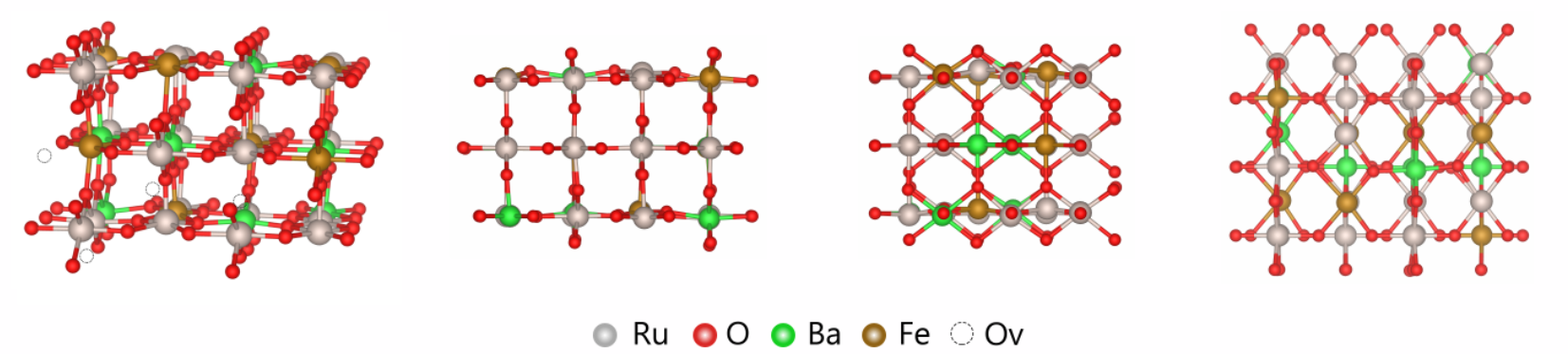


Figure S27. DFT calculation model of BFRO.


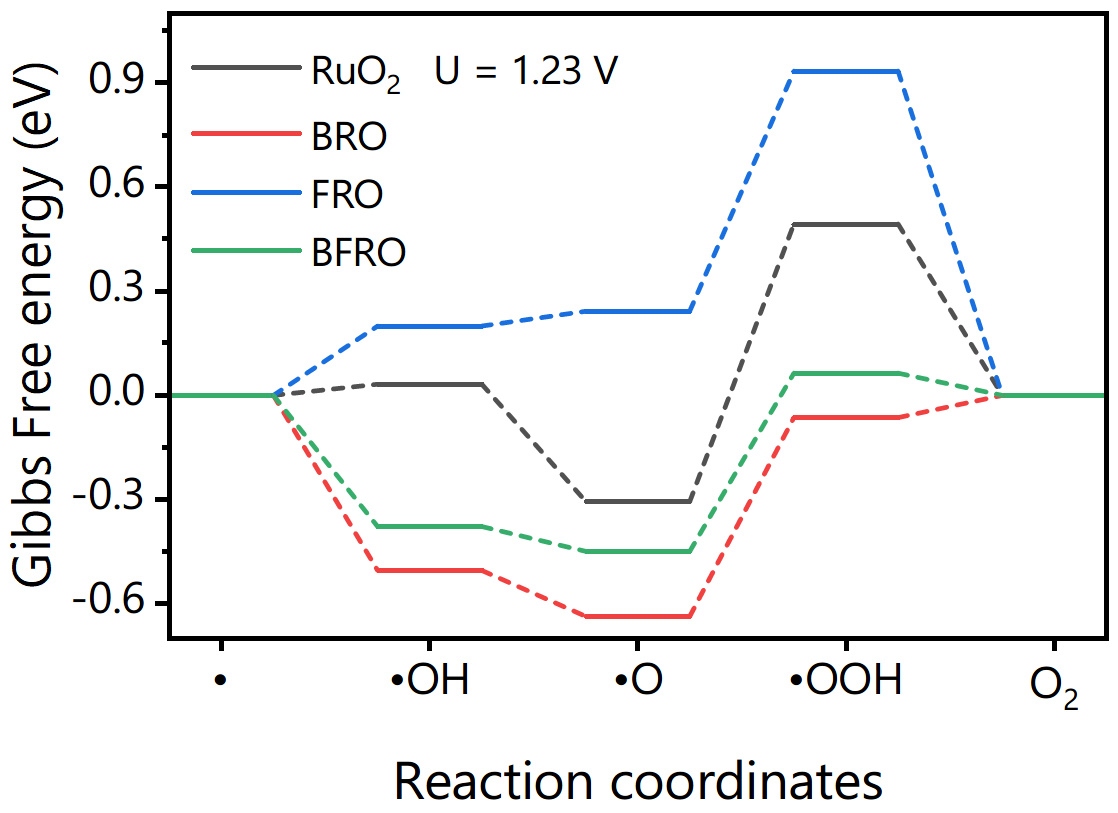


Figure S28. Gibbs free energy change during OER at 1.23 V.


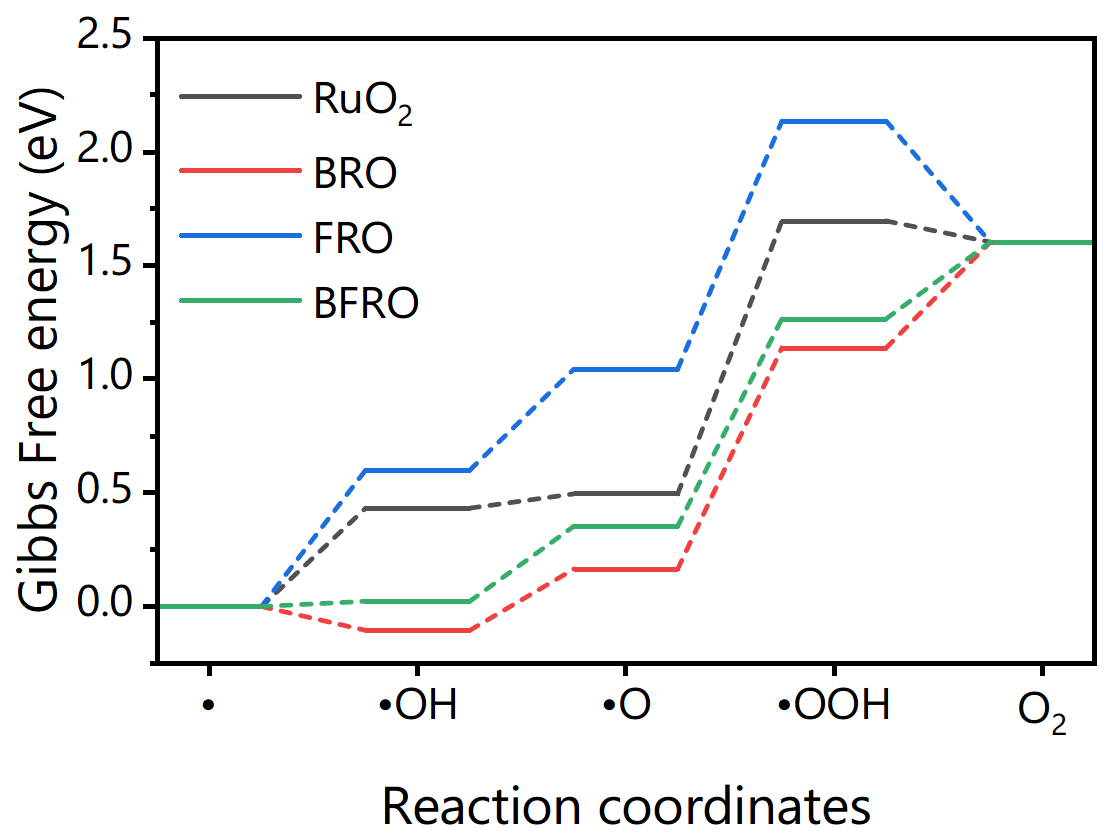


Figure S29. Gibbs free energy change in alkaline electrolyte.


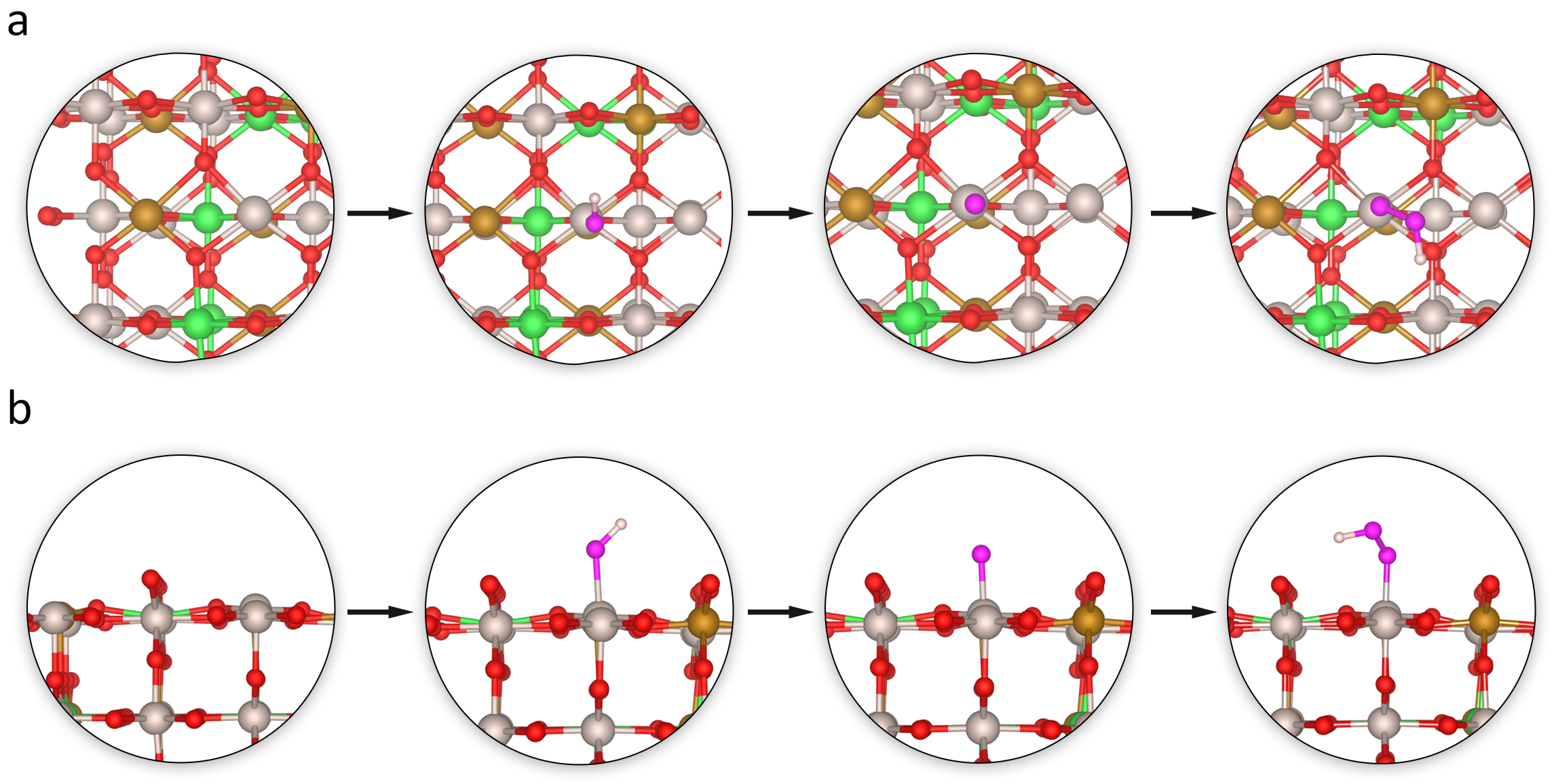


Figure S30. Schematic representation of the OER reaction coordinates on BFRO: (a) top view and (b) front view.


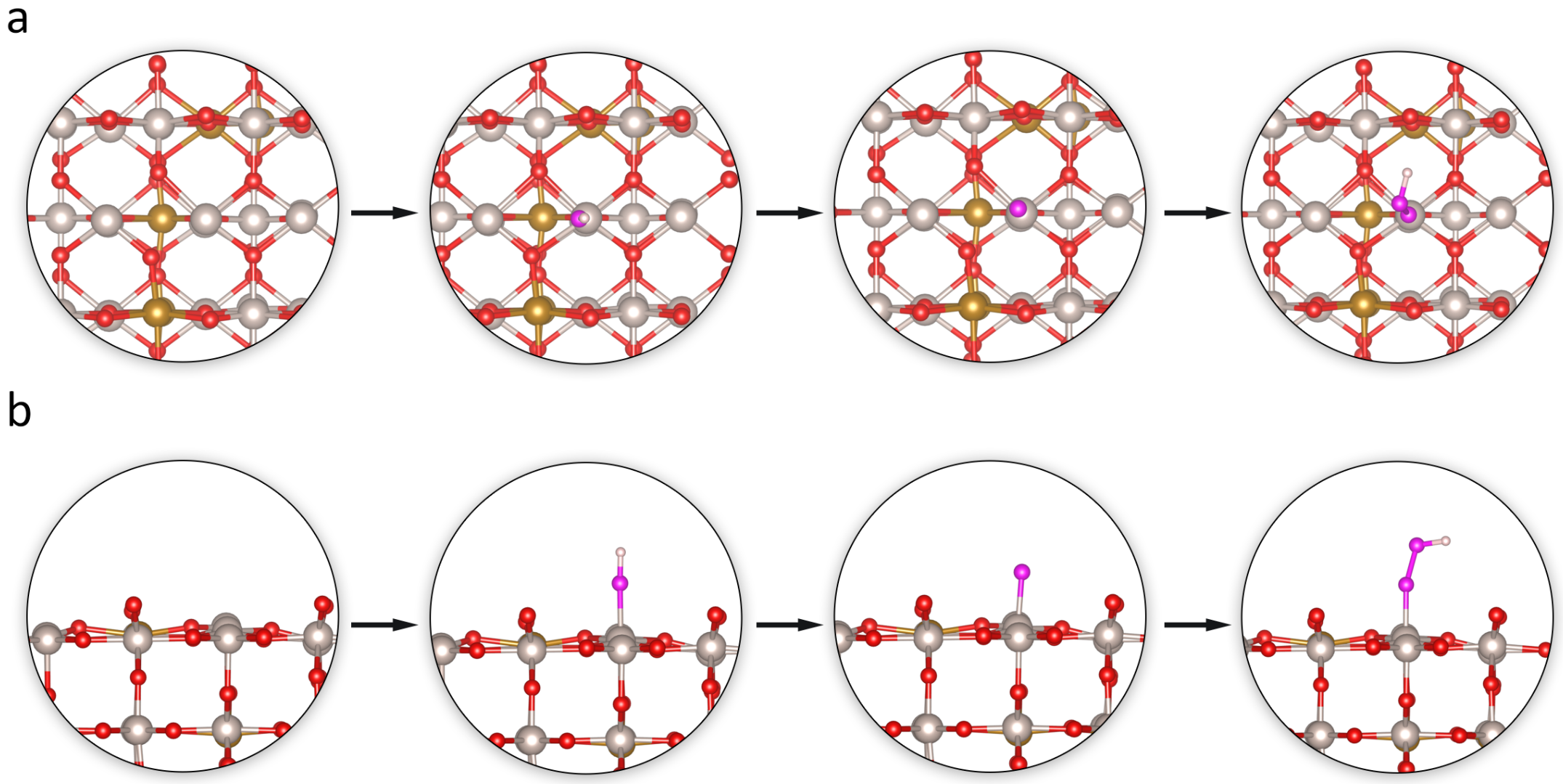


Figure S31. Schematic representation of the OER reaction coordinates on FRO: (a) top view and (b) front view.


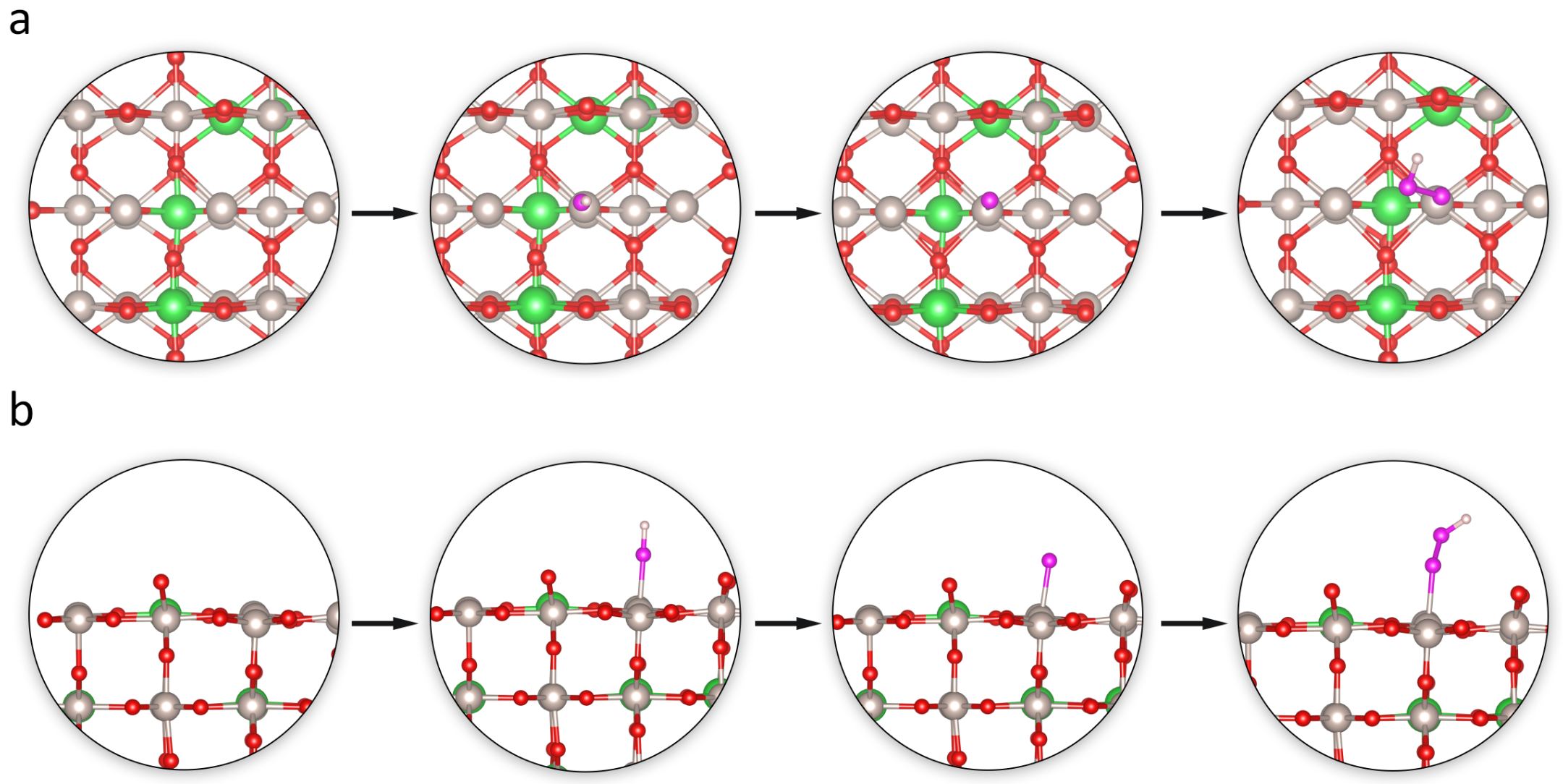


Figure S32. Schematic representation of the OER reaction coordinates on BRO: (a) top view and (b) front view.


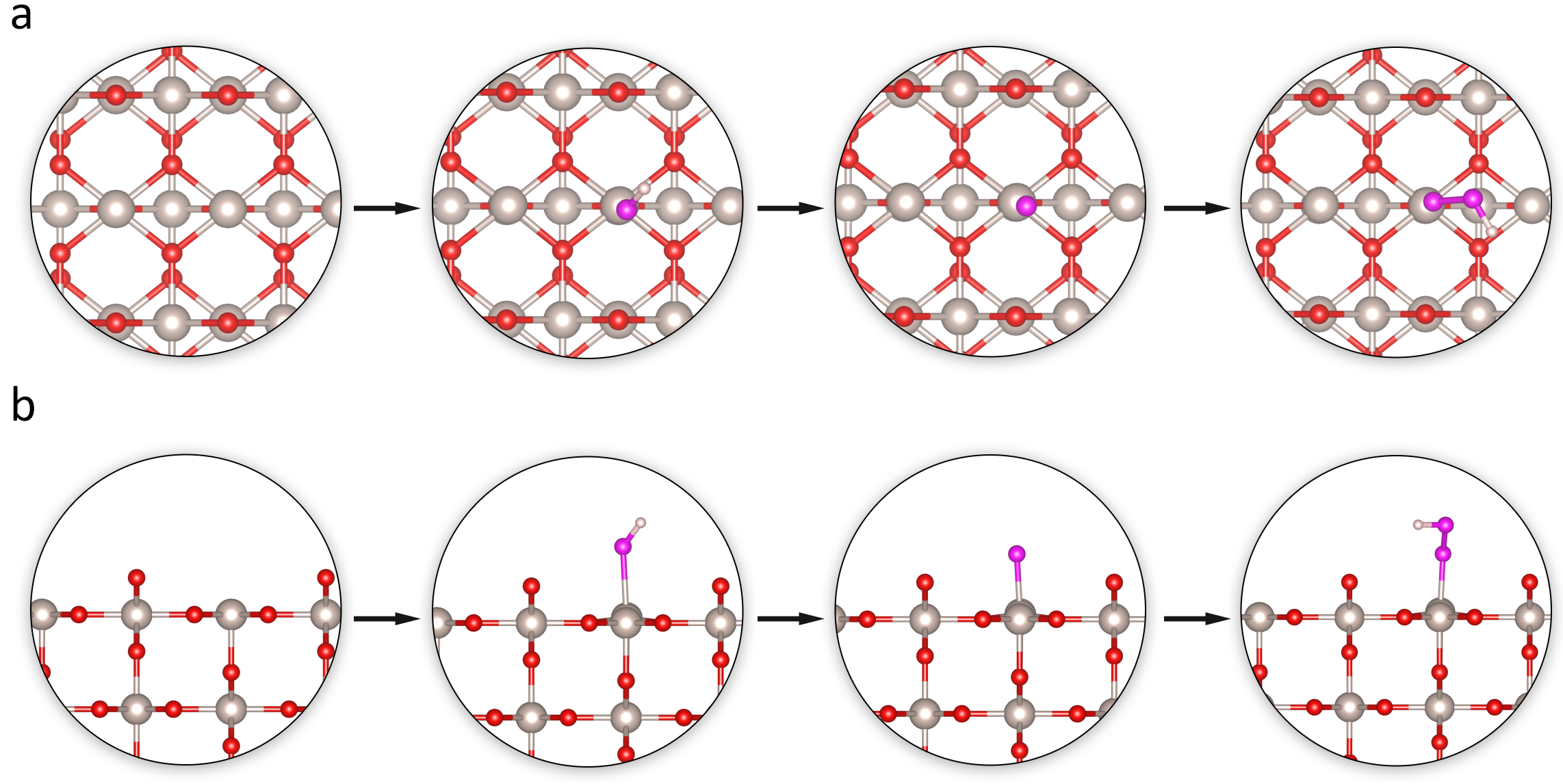


Figure S33. Schematic representation of the OER reaction coordinates on RuO_2_: (a) top view and (b) front view.


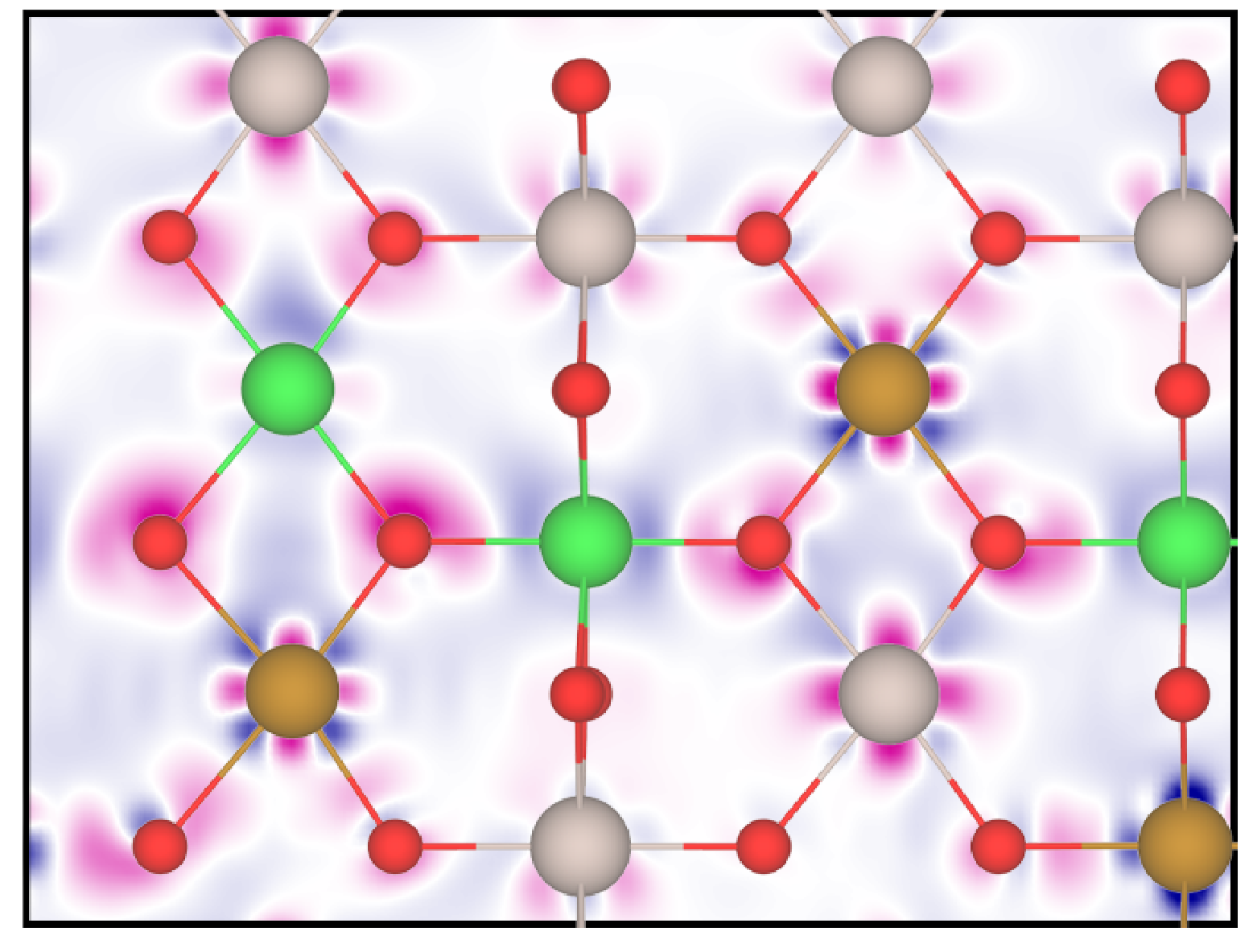


Figure S34. Differential charge density diagram of BFRO on a cross section perpendicular to [110] direction.


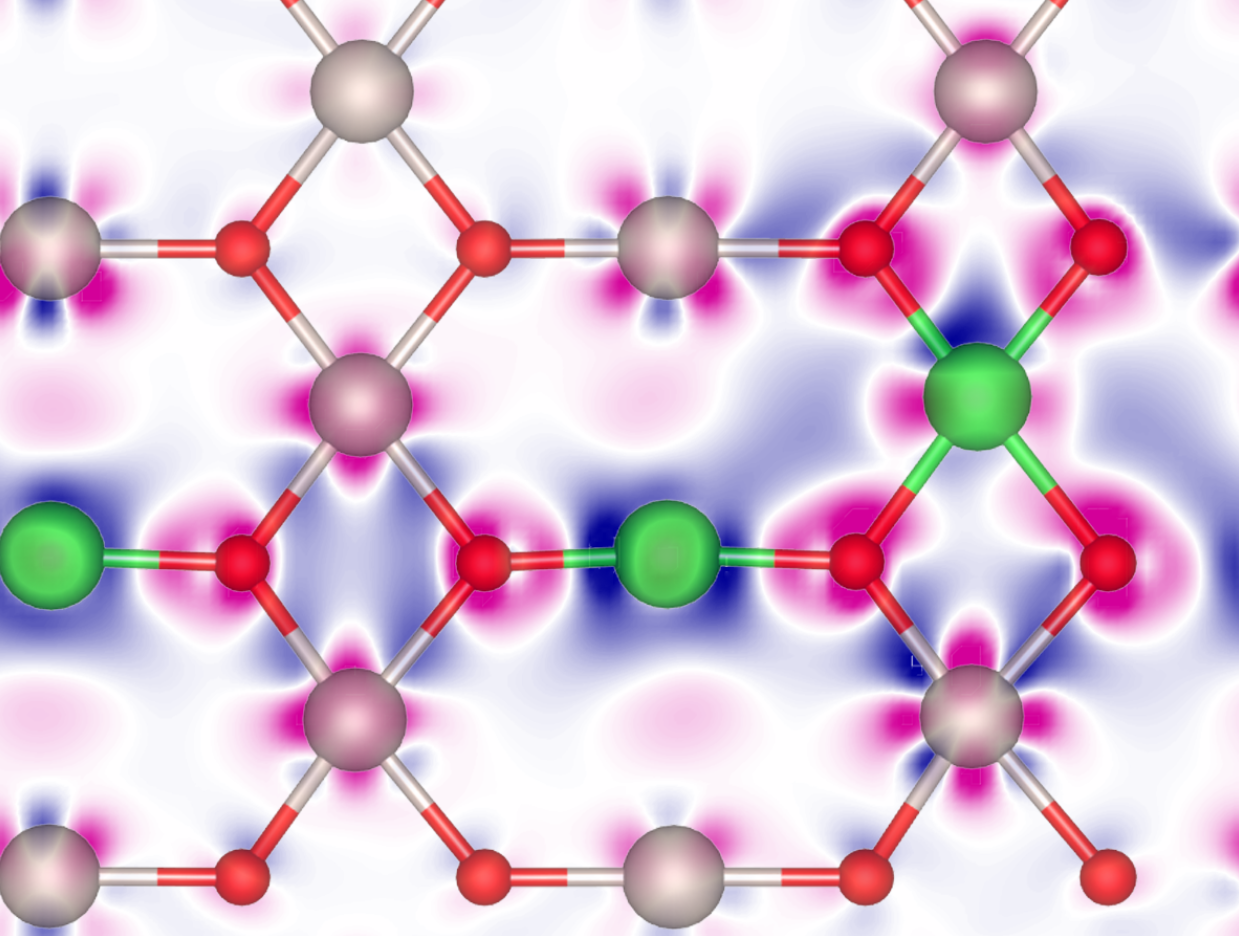


Figure S35. Differential charge density diagram of BRO on a cross section perpendicular to [110] direction.


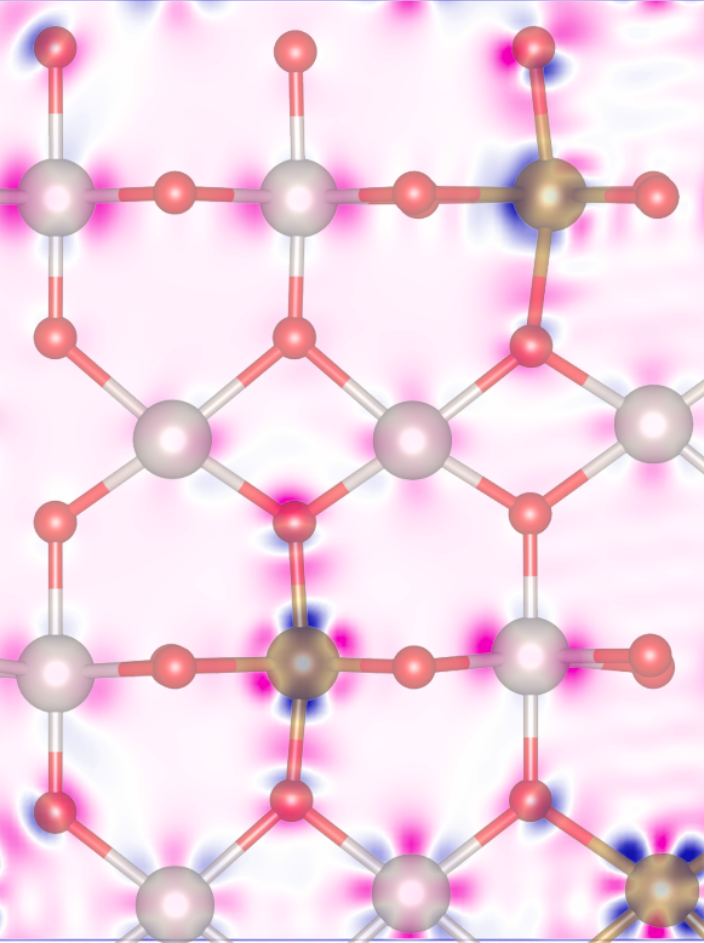


Figure S36. Differential charge density diagram of FRO on a cross section perpendicular to [110] direction.
